# Some Results for Double Cyclic Codes over $F_{q}+vF_{q}+v^{2}F_{q}$

**DOI:** 10.3390/e26090803

**Published:** 2024-09-20

**Authors:** Tenghui Deng, Jing Yang

**Affiliations:** 1 Department of Mathematics, Tsinghua University, Beijing 100084, China; dth16@tsinghua.org.cn; 2 College of Mathematics Science, Inner Mongolia Normal University, Hohhot 010022, China; 3 Center for Applied Mathematical Science, Inner Mongolia, Hohhot 010022, China; 4 Laboratory of Infinite-Dimensional Hamiltonian System and Its Algorithm Application, Inner Mongolia, Hohhot 010022, China

**Keywords:** double cyclic codes, non-chain rings, generator polynomials, generator matrices, 94B05, 94B15

## Abstract

Let $F_{q}$ be a finite field with an odd characteristic. In this paper, we present a new result about double cyclic codes over a finite non-chain ring. Specifically, we study the double cyclic code over $F_{q}+vF_{q}+v^{2}F_{q}$ with $v^{3}=v$, which is isomorphic to $F_{q}\times F_{q}\times F_{q}$. This study mainly involves generator polynomials and generator matrices. The generating polynomial of the dual code is also obtained. We show the relationship between the generating polynomials of the double cyclic codes and those of their dual codes. Finally, as an application of these results, we construct some optimal codes over $F_{3}$.

## 1. Introduction

The theory of error-correcting codes plays a crucial role in Internet data transmission, satellite positioning, and communication. The significance has become increasingly prominent with the promotion and popularization of high technologies such as artificial intelligence and 5G technology. For most coding researchers, cyclic codes are the leading research object because of their reasonable structure and their ease of discovery, understanding, and decoding in the application process.

Coding theory over finite rings is gaining increasing attention in current research due to the increased flexibility of algebraic structures. Recently, Borges et al. proposed a new class of codes called $Z_{2}Z_{4}$-additive codes in [1]. Since then, the theory and applications of this code family have been substantial, and various generalizations have received extensive research. Works by Abualrub in [2,3] and Aydogdu in [4,5,6] have been remarkable. Around 2014, Borges et al. investigated the algebraic structures of double cyclic codes over $Z_{2}$ in [7]. The generating polynomials and duals for this family of codes were identified by the authors. It was vital work for the research on double cyclic codes. Since then, numerous articles about double cyclic codes have appeared, such as [8,9,10,11]. Additionally, references like [12,13,14,15,16] are also relevant to this topic. In particular, references to double skew cyclic codes over $F_{q}$ ([17]) and $F_{q^{2}}$-double cyclic codes concerning the Hermitian inner product ([18]) represent generalized theories of double cyclic codes over finite fields. For $F_{q}+vF_{q}+v^{2}F_{q}$ with $v^{3}=v$, there are many results about this non-chain ring, such as [19,20,21].

In this paper, by applying the methods developed by Borges et al. and Gao et al., we obtain the algebraic structures of double cyclic codes over $F_{q}+vF_{q}+v^{2}F_{q}$. Specifically, we give some examples of these double cyclic codes that improve upon those available in the database [22]. The main results of this paper are listed as follows.

**Theorem 1.**  *Let C be an R-double cyclic code of length (m,n) over $F_{q}+vF_{q}+v^{2}F_{q}$.*

*Then, $C,C^{⊥}$ have the forms of*

$$\left\{\begin{array}{cc} C & =\langle (\iota (x)|0),(\ell (x)|\theta (x))\rangle =\langle \left(\sum_{i=1}^{3}\iota_{v_{i}}\left(x\right)v_{i}|0\right),\left(\sum_{i=1}^{3}\ell_{v_{i}}\left(x\right)v_{i}|\sum_{i=1}^{3}\theta_{v_{i}}\left(x\right)v_{i}\right)\rangle ,\\ C^{⊥} & =\langle \left(\bar{\iota }\left(x\right)|0\right),\left(\bar{\ell }\left(x\right)|\bar{\theta }\left(x\right)\right)\rangle =\langle \left(\sum_{i=1}^{3}{\bar{\iota }}_{v_{i}}\left(x\right)v_{i}|0\right),\left(\sum_{i=1}^{3}{\bar{\ell }}_{v_{i}}\left(x\right)v_{i}|\sum_{i=1}^{3}{\bar{\theta }}_{v_{i}}\left(x\right)v_{i}\right)\rangle . \end{array}\right.$$

*where $\iota_{v_{i}}\left(x\right)|x^{m}-1$, $\theta_{v_{i}}\left(x\right)|x^{n}-1$, and i=1,2,3.*

*If C is a separable R-double cyclic code, we have $\ell_{v_{i}}\left(x\right)=0,i=1,2,3$.*

*If C is a free R-double cyclic code, then the following statements hold:*

*(1). $\deg\left(\ell_{v_{i}}\left(x\right)\right)<\deg\left(\iota_{v_{i}}\left(x\right)\right),i=1,2,3$*

*(2). $\iota_{v_{i}}\left(x\right)|\frac{x^{n}-1}{\theta_{v_{i}}\left(x\right)}\gcd\left(\iota_{v_{i}}\left(x\right),\ell_{v_{i}}\left(x\right)\right),i=1,2,3$*

**Theorem 2.**  *Let C be a double cyclic code of length (m,n) over $F_{q}+vF_{q}+v^{2}F_{q}$, and set $C,C^{⊥}$ to have the forms of*

$$\left\{\begin{array}{cc} C & =\langle (\iota (x)|0),(\ell (x)|\theta (x))\rangle =\langle \left(\sum_{i=1}^{3}\iota_{v_{i}}\left(x\right)v_{i}|0\right),\left(\sum_{i=1}^{3}\ell_{v_{i}}\left(x\right)v_{i}|\sum_{i=1}^{3}\theta_{v_{i}}\left(x\right)v_{i}\right)\rangle ,\\ C^{⊥} & =\langle \left(\bar{\iota }\left(x\right)|0\right),\left(\bar{\ell }\left(x\right)|\bar{\theta }\left(x\right)\right)\rangle =\langle \left(\sum_{i=1}^{3}{\bar{\iota }}_{v_{i}}\left(x\right)v_{i}|0\right),\left(\sum_{i=1}^{3}{\bar{\ell }}_{v_{i}}\left(x\right)v_{i}|\sum_{i=1}^{3}{\bar{\theta }}_{v_{i}}\left(x\right)v_{i}\right)\rangle . \end{array}\right.$$

*Then,*

(1) $\bar{\iota }\left(x\right)=\sum_{i=1}^{3}\frac{x^{m}-1}{\gcd^{*}\left(\iota_{v_{i}}\left(x\right),\ell_{v_{i}}\left(x\right)\right)}v_{i}$

(2) $\bar{\theta }\left(x\right)=\sum_{i=1}^{3}\frac{\left(x^{n}-1\right)\gcd^{*}\left(\iota_{v_{i}}\left(x\right),\ell_{v_{i}}\left(x\right)\right)}{\iota_{v_{i}}^{*}\left(x\right)\theta_{v_{i}}^{*}\left(x\right)}v_{i}$

(3) $\bar{\ell }\left(x\right)=\rho \left(x\right)\sum_{i=1}^{3}\frac{x^{m}-1}{\iota_{v_{i}}^{*}\left(x\right)}v_{i}$, where

$$\left\{\begin{array}{c} \rho (x)=0\;\mathrm{if}\;C\;\mathrm{is}\;\mathrm{separable},\;\mathrm{or}\;\mathrm{otherwise}\\ \rho \left(x\right)=\left(\sum_{i=1}^{3}-x^{l-\deg\left(\theta_{v_{i}}\left(x\right)\right)+\deg\left(\iota_{v_{i}}\left(x\right)\right)}v_{i}\right){\left(\frac{\iota^{*}\left(x\right)}{\gcd^{*}\left(\iota \left(x\right),\ell \left(x\right)\right)}\right)}^{-1}mod\frac{\iota^{*}\left(x\right)}{\gcd^{*}\left(\iota \left(x\right),\ell \left(x\right)\right)}. \end{array}\right.$$

The rest of the article is organized as follows. In Section 2, we provide some necessary preliminaries about the polynomial theory over $Fq+vFq+v^{2}Fq$. In Section 3, we present the definition of double cyclic codes and set up some structural properties of double cyclic codes over $Fq+vFq+v^{2}Fq$. Section 4 gives the generating matrix forms of these double cyclic codes. Simultaneously, utilizing these generating matrix forms, we obtain some quantitative information relating to double cyclic codes. Section 5 discusses the relationship between the generators of double cyclic codes and their duals. In Section 6, some examples of optimal codes over finite fields are given.

## 2. Preliminaries

Throughout this paper, let *R* denote $F_{q}+vF_{q}+v^{2}F_{q}$, where $v^{3}=v$ and $F_{q}$ is a finite field of odd characteristic. From the theory of finite rings, we know that *R* is isomorphic to the quotient ring $F_{q}\left[x\right]/\langle v^{3}-v\rangle$, which is a finite commutative ring with identity. This implies that *R* is a principal ring with exactly three non-trivial maximal ideals: $\langle 1-v\rangle ,\;\langle v\rangle ,\;\langle 1+v\rangle$. Consequently, by the Chinese Remainder Theorem, we obtain

$$R=R/\langle v\rangle ⊕R/\langle 1-v\rangle ⊕R/\langle 1+v\rangle .$$

Define $v_{1}=1-v^{2},\;v_{2}=\frac{v+v^{2}}{2},\;v_{3}=\frac{v^{2}-v}{2}$. Note that $v_{1},v_{2},v_{3}$ are orthogonal idempotent elements in *R*. Thus, *R* can be decomposed as

$$R=Rv_{1}⊕Rv_{2}⊕Rv_{3}=F_{q}v_{1}⊕F_{q}v_{2}⊕F_{q}v_{3}.$$

For r∈R, express *r* as $r=\sum_{i=1}^{3}r_{v_{i}}v_{i}$ with $r_{v_{i}}\in F_{q},\;\mathrm{for}\;i=1,2,3$. Define three projections $P_{v_{i}}:r=\sum_{i=1}^{3}r_{v_{i}}v_{i}\mapsto r_{v_{i}},i=1,2,3$. These mappings $P_{v_{i}},i=1,2,3$ are $F_{q}$-algebra homomorphisms. For any n∈N, extend $P_{v_{i}}$ naturally from *R* to $R^{n}$ as $P_{v_{i}}:R^{n}\to F_{q}^{n},\;\left(r_{1},\ldots r_{n}\right)\mapsto \left({\left(r_{1}\right)}_{v_{i}},\ldots {\left(r_{n}\right)}_{v_{i}}\right)$, i=1,2,3. These extensions remain $F_{q}$-algebra homomorphisms, and similarly extend to polynomial rings over *R*.

For each polynomial r(x)∈R[x], considering the commutativity with $\left\{v_{1},v_{2},v_{3}\right\}$ and *x*, decompose the coefficients into standard bases, and appropriately combine analogous terms to obtain the unique decomposition $r\left(x\right)=\sum_{i=1}^{3}r_{v_{i}}\left(x\right)v_{i}$ relative to these standard bases. Define three maps $P_{v_{i}}:R\left[x\right]\to F_{q}\left[x\right]\;r\left(x\right)=\sum_{i=1}^{3}r_{v_{i}}\left(x\right)v_{i}\mapsto r_{v_{i}}\left(x\right),i=1,2,3$. For a(x),b(x)∈R[x], let $a\left(x\right)=\sum_{i=1}^{3}a_{v_{i}}\left(x\right)v_{i},\;b\left(x\right)=\sum_{i=1}^{3}b_{v_{i}}\left(x\right)v_{i}$, and we have that

$$\left\{\begin{array}{cc} a(x)+b(x) & =\sum_{i=1}^{3}\left(a_{v_{i}}\left(x\right)+b_{v_{i}}\left(x\right)\right)v_{i},\\ a(x)b(x) & =\sum_{i=1}^{3}\left(a_{v_{i}}\left(x\right)b_{v_{i}}\left(x\right)\right)v_{i}. \end{array}\right.$$

This means that the projections $P_{v_{i}},i=1,2,3$ into R[x] are also $F_{q}\left[x\right]$-homomorphisms. From the above station, we obtain that $R\left[x\right]=\left({⊕}_{i=1}^{3}F_{q}v_{i}\right)\left[x\right]={⊕}_{i=1}^{3}F_{q}\left[x\right]v_{i}$.

Regarding the divisibility between any two elements in R[x], we have the following.

**Lemma 1.**  *Let a(x),b(x)∈R[x] with $a\left(x\right)=\sum_{i=1}^{3}a_{v_{i}}\left(x\right)v_{i},b\left(x\right)=\sum_{i=1}^{3}b_{v_{i}}\left(x\right)v_{i}$. Then, a(x)|b(x) in R[x], if and only if $a_{v_{i}}\left(x\right)|b_{v_{i}}\left(x\right)$, i=1,2,3 in $F_{q}\left[x\right]$.*

**Proof.**  If a(x)|b(x), we can set b(x)=e(x)a(x) with e(x)∈R[x]. Hence,

$$\sum_{i=1}^{3}b_{v_{i}}\left(x\right)v_{i}=\left(\sum_{i=1}^{3}e_{v_{i}}\left(x\right)v_{i}\right)\left(\sum_{i=1}^{3}a_{v_{i}}\left(x\right)v_{i}\right)=\sum_{i=1}^{3}\left(e_{v_{i}}\left(x\right)a_{v_{i}}\left(x\right)\right)v_{i}.$$

Then, we have $b_{v_{i}}\left(x\right)=e_{v_{i}}\left(x\right)a_{v_{i}}\left(x\right),i=1,2,3$. This means that $a_{v_{i}}\left(x\right)|b_{v_{i}}\left(x\right),i=1,2,3$ in $F_{q}\left[x\right]$.

On the contrary, due to $a_{v_{i}}\left(x\right)|b_{v_{i}}\left(x\right),i=1,2,3$, we can set $b_{v_{i}}\left(x\right)=e_{v_{i}}\left(x\right)a_{v_{i}}\left(x\right)$ with $e_{v_{i}}\left(x\right)\in F_{q}\left[x\right]$i=1,2,3. Then,

$$b\left(x\right)=\sum_{i=1}^{3}b_{v_{i}}\left(x\right)v_{i}=\sum_{i=1}^{3}e_{v_{i}}\left(x\right)a_{v_{i}}\left(x\right)v_{i}=\left(\sum_{i=1}^{3}e_{v_{i}}\left(x\right)v_{i}\right)\left(\sum_{i=1}^{3}a_{v_{i}}\left(x\right)v_{i}\right)=\left(\sum_{i=1}^{3}e_{v_{i}}\left(x\right)v_{i}\right)a\left(x\right).$$

Let $e\left(x\right)=\sum_{i=1}^{3}e_{v_{i}}\left(x\right)v_{i}\in R\left[x\right]$. This shows that a(x)|b(x) in R[x]. □

**Remark 1.**  *From Lemma 1, it also follows that $P_{v_{i}}\left(\frac{b(x)}{a(x)}\right)=e_{v_{i}}\left(x\right)=\frac{P_{v_{i}}\left(b\left(x\right)\right)}{P_{v_{i}}\left(a\left(x\right)\right)}$, i=1,2,3, when a(x),b(x)∈R[x], a(x)|b(x).*

We also have the following finding on the greatest common divisor between any two elements in R[x] for the purpose of the divisible form.

**Lemma 2.**  *Let a(x),b(x)∈R[x] with $a\left(x\right)=\sum_{i=1}^{3}a_{v_{i}}\left(x\right)v_{i},b\left(x\right)=\sum_{i=1}^{3}b_{v_{i}}\left(x\right)v_{i}$. Then, in R[x], we have*

$$\gcd\left(a\left(x\right),b\left(x\right)\right)=\gcd\left(\sum_{i=1}^{3}a_{v_{i}}\left(x\right)v_{i},\sum_{i=1}^{3}b_{v_{i}}\left(x\right)v_{i}\right)=\sum_{i=1}^{3}\gcd\left(a_{v_{i}}\left(x\right),b_{v_{i}}\left(x\right)\right)v_{i},$$

*where the greatest common divisor of the polynomials in $F_{q}\left[x\right]$ is denoted by the gcd(−,−) on the right side of the expression.*

**Proof.**  Denote $\sum_{i=1}^{3}\gcd\left(a_{v_{i}}\left(x\right),b_{v_{i}}\left(x\right)\right)v_{i}$ by m(x), then m(x)∈R[x]. From the conventional polynomial theory, we know that $\gcd\left(a_{v_{i}}\left(x\right),b_{v_{i}}\left(x\right)\right)|a_{v_{i}}\left(x\right)$, $\gcd\left(a_{v_{i}}\left(x\right),b_{v_{i}}\left(x\right)\right)|b_{v_{i}}\left(x\right)$, i=1,2,3. For Lemma 1, we have m(x)|a(x), m(x)|b(x). It follows that m(x) is a common divisor of a(x) and b(x).

Let r(x)∈R[x] with r(x)|a(x), r(x)|b(x). We can write $r\left(x\right)=\sum_{i=1}^{3}r_{v_{i}}\left(x\right)v_{i}$. By the same reasoning as in Lemma 1, we have $r_{v_{i}}\left(x\right)|a_{v_{i}}\left(x\right)$, $r_{v_{i}}\left(x\right)|b_{v_{i}}\left(x\right)$ for i=1,2,3. Thus, $r_{v_{i}}\left(x\right)|\gcd\left(a_{v_{i}}\left(x\right),b_{v_{i}}\left(x\right)\right)$ in $F_{q}\left[x\right]$ for i=1,2,3. Consequently, r(x)|m(x). This reveals that every common divisor of a(x) and b(x) is a divisor of m(x).

In conclusion, we have $\sum_{i=1}^{3}\gcd\left(a_{v_{i}}\left(x\right),b_{v_{i}}\left(x\right)\right)v_{i}=m\left(x\right)=\gcd\left(a\left(x\right),b\left(x\right)\right)$. □

**Remark 2.**  *From the proof above, we also have*

$$P_{v_{i}}\left(\gcd\left(a\left(x\right),b\left(x\right)\right)\right)=m_{v_{i}}\left(x\right)=\gcd\left(P_{v_{i}}\left(a\left(x\right)\right),P_{v_{i}}\left(b\left(x\right)\right)\right),i=1,2,3.$$

Summarizing the above statement, we have

$$R\left[x\right]/\langle r[x]\rangle =\left({⊕}_{i=1}^{3}F_{q}\left[x\right]v_{i}\right)/\langle \sum_{i=1}^{3}r_{v_{i}}\left(x\right)v_{i}\rangle ={⊕}_{i=1}^{3}\left(F_{q}\left[x\right]/\langle r_{v_{i}}\left(x\right)\rangle \right)v_{i},$$

where r(x)∈R[x] with $r\left(x\right)=\sum_{i=1}^{3}r_{v_{i}}\left(x\right)v_{i}$.

For cyclic codes over *R*, see [19].

## 3. Double Cyclic Codes

In this section, we describe the basic structure of *R*-double cyclic codes.

**Definition 1.**  *Let C be an R-linear code of length m+n. A code C is referred to as a double cyclic code of length (m,n) over R if, for any*

$$c=(c_{0}^{1},\cdots ,c_{m-2}^{1},c_{m-1}^{1}|c_{0}^{2},\cdots ,c_{n-2}^{2},c_{n-1}^{2})\in C,$$

*it implies the cyclic shift*

$$T\left(c\right)=\left(c_{m-1}^{1},c_{0}^{1},\cdots ,c_{m-2}^{1}|c_{n-1}^{2},c_{0}^{2},\cdots ,c_{n-2}^{2}\right)\in C.$$

We learn that the double cyclic code *C* can be thought of as an *R*-submodule of $R^{m}\times R^{n}$ from the definition of double cyclic codes over *R*.

Let $c,d\in R^{m}\times R^{n}$ with $c=\left(c_{0}^{1},\cdots ,c_{m-1}^{1}|c_{0}^{2},\cdots ,c_{n-1}^{2}\right),d=\left(d_{0}^{1},\cdots ,d_{m-1}^{1}|d_{0}^{2},\cdots ,d_{n-1}^{2}\right)$; we define the inner product of these two elements as $\langle c,d\rangle ≜\sum_{i=0}^{m-1}c_{i}^{1}d_{i}^{1}+\sum_{j=0}^{n-1}c_{j}^{2}d_{j}^{2}$.

For the double cyclic code *C*, the dual code is defined as

$$C^{⊥}=\left\{d\in R^{m}\times R^{n}|\langle d,c\rangle =0,\;\forall c\in C\right\}.$$

For $R^{m}\times R^{n}$, define two coordinate projections as

$$\left\{\begin{array}{cc} P_{m}:R^{m}\times R^{n}\to R^{m}, & \left(r_{1}^{1},\cdots ,r_{m}^{1}|r_{1}^{2},\cdots ,r_{n}^{2}\right)\mapsto \left(r_{1}^{1},\cdots ,r_{m}^{1}\right),\\ P_{n}:R^{m}\times R^{n}\to R^{n}, & \left(r_{1}^{1},\cdots ,r_{m}^{1}|r_{1}^{2},\cdots ,r_{n}^{2}\right)\mapsto \left(r_{1}^{2},\cdots ,r_{n}^{2}\right). \end{array}\right.$$

For $f\left(x\right)\in F_{q}\left[x\right]$, owing to $\sum_{i=1}^{3}v_{i}=1$, we can view it as $f\left(x\right)=\sum_{i=1}^{3}f\left(x\right)v_{i}$. This guarantees that, for every $f\left(x\right)\in F_{q}\left[x\right]$, the quotient ring $R\left[x\right]/\langle f(x)\rangle$ is well-defined. Then, for $x^{m}-1,x^{n}-1\in F_{q}\left[x\right]$, let

$$R_{m}\left[x\right]≜R\left[x\right]/\langle x^{m}-1\rangle ;R_{m,n}\left[x\right]≜\left(R\left[x\right]/\langle x^{m}-1\rangle \right)\times \left(R\left[x\right]/\langle x^{n}-1\rangle \right);R_{n}\left[x\right]≜R\left[x\right]/\langle x^{n}-1\rangle .$$

The multiplication of R[x] can induce the action of R[x] on $R_{m}\left[x\right]$, $R_{m,n}\left[x\right]$ and $R_{n}\left[x\right]$ naturally. With this action, the rings $R_{m}\left[x\right]$, $R_{m,n}\left[x\right]$, and $R_{n}\left[x\right]$ become R[x]-modules. Let $r\left(x\right)\in R\left[x\right],a\left(x\right)\in R_{m}\left[x\right],b\left(x\right)\in R_{n}\left[x\right]$, then $\left(a\left(x\right)|b\left(x\right)\right)\in R_{m,n}\left[x\right]$ and

$$\left\{\begin{array}{c} r\left(x\right)\circ a\left(x\right)=r\left(x\right)a\left(x\right)(\mathrm{mod}\left(x^{m}-1\right)),\\ r\left(x\right)\circ \left(a\left(x\right)|b\left(x\right)\right)=(r\left(x\right)a\left(x\right)\left(mod\left(x^{m}-1\right)\right)|r\left(x\right)b\left(x\right)\left(\mathrm{mod}\left(x^{n}-1\right)\right),\\ r\left(x\right)\circ b\left(x\right)=r\left(x\right)b\left(x\right)(\mathrm{mod}\left(x^{n}-1\right)). \end{array}\right.$$

There are also two coordinate projections for the bijection from $R^{m}\times R^{n}$ to $R_{m,n}\left[x\right]$, given by

$$\left(c_{0}^{1},c_{1}^{1},\cdots ,c_{m-1}^{1}|c_{0}^{2},c_{1}^{2},\cdots ,c_{n-1}^{2}\right)\mapsto \left(c_{0}^{1}+c_{1}^{1}x+\cdots +c_{m-1}^{1}x^{m-1}|c_{0}^{2}+c_{1}^{2}x+\cdots +c_{n-1}^{2}x^{n-1}\right).$$

They are

$$\left\{\begin{array}{cc} P_{m}:R_{m,n}\left[x\right]\to R_{m}\left[x\right] & (a(x)|b(x))\mapsto a(x),\\ P_{n}:R_{m,n}\left[x\right]\to R_{n}\left[x\right] & (a(x)|b(x))\mapsto b(x). \end{array}\right.$$

Then, $P_{m},\;P_{n}$ are still R[x]-module homomorphisms. Similarly, based on the one-to-one correspondence between $R^{m}\times R^{n}$ and $R_{m,n}\left[x\right]$, it reveals the fact that *C* is a double cyclic code of length (m,n) over *R*, if and only if the corresponding polynomial set is an R[x]-submodule of $R_{m,n}\left[x\right]$. Then, we can use the R[x]-submodule of $R_{m,n}\left[x\right]$, and regard *R*-double cyclic codes as the R[x]-submodule of $R_{m,n}\left[x\right]$. At the same time, we obtain two types of projections: the canonical projections ($P_{v_{i}}$, i=1,2,3) and the coordinate projections ($P_{m},P_{n}$). Unless otherwise specified, a mathematical object will appear with the subscripts $m,n,v_{1},v_{2},v_{3}$, which means we use their corresponding projections by default.

Let l=[m,n] (i.e., *l* denotes the least common multiple of *m* and *n*).

**Proposition 1.**  *If C is a double cyclic code of length (m,n) over R, then the dual code $C^{⊥}$ is also an R-double cyclic code of the same length.*

**Proof.**  Assume that *C* is an *R*-double cyclic code of length (m,n), and $d=(d_{0}^{1},\cdots ,d_{m-1}^{1}|d_{0}^{2},\cdots ,d_{n-1}^{2})$ is a codeword in $C^{⊥}$. We must describe how the cyclic shift codeword $T\left(d\right)\in C^{⊥}$ is defined for *R*-double cyclic codes. This means that we need to prove $\langle T(d),c\rangle =0$ for all codewords c∈C.

Let *c* be any codeword of *C*. By mathematical induction, we can easily obtain $T^{l-1}\left(c\right)=T^{l-2}\left(T\left(c\right)\right)\in C$. Note that $T^{l}\left(c\right)=c$. Fix $c=(c_{0}^{1},\cdots ,c_{m-1}^{1}|c_{0}^{2},\cdots ,c_{n-1}^{2})$, and write the specific form of $T^{l-1}\left(c\right)$; we obtain $T^{l-1}\left(c\right)=\left(c_{1}^{1},\cdots ,c_{m-1}^{1},c_{0}^{1}|c_{1}^{2},\cdots ,c_{n-1}^{2},c_{0}^{2}\right)$. Since $d\in C^{⊥}$ and c∈C, we have

$$\;0=\langle d,T^{l-1}\left(c\right)\rangle =d_{0}^{1}c_{1}^{1}+\cdots +d_{m-2}^{1}c_{0}^{1}+d_{m-1}^{1}c_{0}^{1}+d_{0}^{2}c_{1}^{2}+\cdots +d_{m-2}^{2}c_{0}^{2}+d_{m-1}^{2}c_{0}^{2}=\langle T(d),c\rangle ,$$

which implies that $T\left(d\right)\in C^{⊥}$. Consequently, $C^{⊥}$ is likewise an *R*-double cyclic code of length (m,n). □

**Proposition 2.**  *Let C be an R-double cyclic code with length (m,n). Then, there exist polynomials $\iota \left(x\right),\ell \left(x\right)\in R_{m}\left[x\right],\theta \left(x\right)\in R_{n}\left[x\right]$ with $\iota \left(x\right)=\sum_{i=1}^{3}\iota_{v_{i}}\left(x\right)v_{i},\ell \left(x\right)=\sum_{i=1}^{3}\ell_{v_{i}}\left(x\right)v_{i},\theta \left(x\right)=\sum_{i=1}^{3}\theta_{v_{i}}\left(x\right)v_{i}$, such that C has the forms of*

$$C=\langle (\iota (x)|0),(\ell (x)|\theta (x))\rangle =\langle \left(\sum_{i=1}^{3}\iota_{v_{i}}\left(x\right)v_{i}|0\right),\left(\sum_{i=1}^{3}\ell_{v_{i}}\left(x\right)v_{i}|\sum_{i=1}^{3}\theta_{v_{i}}\left(x\right)v_{i}\right)\rangle ,$$

*where $\iota_{v_{i}}\left(x\right)|x^{m}-1,i=1,2,3$ and $\theta_{v_{i}}\left(x\right)|x^{n}-1,i=1,2,3$.*

**Proof.**  Consider the coordinate projection $P_{n}:R_{m,n}\left[x\right]\to R_{n}\left[x\right]$ defined by (a(x)|b(x))↦b(x). Clearly, $P_{n}$ is an R[x]-module homomorphism. Since the double cyclic code *C* is an R[x]-submodule of $R_{m,n}\left[x\right]$, $P_{n}\left(C\right)$ is an R[x]-submodule of $R_{n}\left[x\right]$, which means that $P_{n}\left(C\right)$ is an ideal of $R_{n}\left[x\right]$. From the structure of $R_{n}\left[x\right]$, we can write $P_{n}\left(C\right)=\langle \theta (x)\rangle$ with $\theta \left(x\right)=\sum_{i=1}^{3}\theta_{v_{i}}\left(x\right)v_{i}$, where $\theta_{v_{i}}\left(x\right)|\left(x^{n}-1\right)$ for i=1,2,3. Note that $\mathrm{Ker}\left(P_{n}{|}_{C}\right)=\left\{\left(c^{1}\left(x\right)|c^{2}\left(x\right)\right)\in C|c^{2}\left(x\right)=0\right\}$. Define the set $I=\left\{c^{1}\left(x\right)\in R_{m}\left[x\right]|\left(c^{1}\left(x\right)|0\right)\in \mathrm{Ker}\left(P_{n}{|}_{C}\right)\right\}$. Obviously, *I* is an ideal of $R_{m}\left[x\right]$. Similarly, $I=\langle \iota (x)\rangle$ with $\iota \left(x\right)=\sum_{i=1}^{3}\iota_{v_{i}}\left(x\right)v_{i}$, where $\iota_{v_{i}}\left(x\right)|\left(x^{m}-1\right)$ for i=1,2,3.

For any element $\left(c^{1}\left(x\right)|0\right)\in \mathrm{Ker}\left(P_{n}{|}_{C}\right)$, there exists a polynomial m(x)∈R[x], such that $c^{1}\left(x\right)=m\left(x\right)\iota \left(x\right)$. Thus, $\left(c^{1}\left(x\right)|0\right)=m\left(x\right)\left(\iota \left(x\right)|0\right)$, implying that $\mathrm{Ker}(P_{n}{|}_{C})$ is an R[x]-submodule of *C* generated by (ι(x)|0). By the first isomorphism theorem for module homomorphisms, we have $C/\mathrm{Ker}\left(P_{n}{|}_{C}\right)≅\mathrm{Img}\left(P_{n}{|}_{C}\right)=P_{n}\left(C\right)=\langle \theta (x)\rangle$.

For θ(x), let (ℓ(x)|θ(x))∈C with $P_{n}\left(\ell \left(x\right)|\theta \left(x\right)\right)=\theta \left(x\right)$, where $\ell \left(x\right)=\sum_{i=1}^{3}\ell_{v_{i}}\left(x\right)v_{i}$, $\ell_{v_{i}}\left(x\right)\in F_{q}\left[x\right]$ for i=1,2,3. In the proof that follows, we demonstrate $C=\langle (\iota (x)|0),$$(\ell (x)|\theta (x))\rangle$.

Let $c\left(x\right)=(c^{1}\left(x\right)|c^{2}\left(x\right))\in C$; then, $c^{2}\left(x\right)=P_{n}\left(\left(c^{1}\left(x\right)|c^{2}\left(x\right)\right)\right)\in P_{n}\left(C\right)$. Therefore, $\exists s\left(x\right)\in R_{n}\left[x\right]$, such that $c^{2}\left(x\right)=s\left(x\right)\theta \left(x\right)$. Hence,

$$c\left(x\right)-s\left(x\right)\left(\ell \left(x\right)|\theta \left(x\right)\right)=\left(c^{1}\left(x\right)|c^{2}\left(x\right)\right)-s\left(x\right)\left(\ell \left(x\right)|\theta \left(x\right)\right)=\left(c^{1}\left(x\right)-s\left(x\right)\ell \left(x\right)|0\right)\in \mathrm{Ker}\left(P_{n}{|}_{C}\right).$$

Thus, $\exists r\left(x\right)\in R_{m}\left[x\right]$, such that $c^{1}\left(x\right)-s\left(x\right)\ell \left(x\right)=r\left(x\right)\iota \left(x\right)$ and $\left(c^{1}\left(x\right)-s\left(x\right)\ell \left(x\right)|0\right)=r\left(x\right)\left(\iota \left(x\right)|0\right)$. Hence,

$$c\left(x\right)=\left(c^{1}\left(x\right)|c^{2}\left(x\right)\right)=r\left(x\right)\left(\iota \left(x\right)|0\right)+s\left(x\right)\left(\ell \left(x\right)|\theta \left(x\right)\right).$$

These indicate that *C* is finitely generated by $\left\{(\iota (x)|0),(\ell (x)|\theta (x))\right\}$. □

**Lemma 3.**  *Let C be an R-double cyclic code with length (m,n), with*

$$C=\langle (\iota (x)|0),(\ell (x)|\theta (x))\rangle =\langle \left(\sum_{i=1}^{3}\iota_{v_{i}}\left(x\right)v_{i}|0\right),\left(\sum_{i=1}^{3}\ell_{v_{i}}\left(x\right)v_{i}|\sum_{i=1}^{3}\theta_{v_{i}}\left(x\right)v_{i}\right)\rangle .$$

*As the most basic forms of generator polynomials, we have $\deg\left(\ell_{v_{i}}\left(x\right)\right)<\deg\left(\iota_{v_{i}}\left(x\right)\right)$ for i=1,2,3.*

**Proof.**  If not, there exists i∈{1,2,3}, such that $\deg\left(\ell_{v_{i}}\left(x\right)\right)\geq \deg\left(\iota_{v_{i}}\left(x\right)\right)$. Without loss of generality, assume $\deg\left(\ell_{v_{3}}\left(x\right)\right)\geq \deg\left(\iota_{v_{3}}\left(x\right)\right)$. Let $k=\deg\left(\ell_{v_{3}}\left(x\right)\right)-\deg\left(\iota_{v_{3}}\left(x\right)\right)$, where k≥0. Define $D=\langle \left(\sum_{i=1}^{3}\iota_{v_{i}}\left(x\right)v_{i}|0\right),\left(\sum_{i=1}^{2}\ell_{v_{i}}\left(x\right)v_{i}+\left(\ell_{v_{3}}\left(x\right)-x^{k}\iota_{v_{3}}\left(x\right)\right)v_{3}|\sum_{i=1}^{3}\theta_{v_{i}}\left(x\right)v_{i}\right)\rangle$. It is obvious that $\deg\left(\ell_{v_{3}}\left(x\right)-x^{k}\iota_{v_{3}}\left(x\right)\right)<\deg\left(\ell_{v_{3}}\left(x\right)\right)$. Since the generators of *D* belong to *C*, we have D⊂C. On the other hand,

$$\left(\sum_{i=1}^{3}\ell_{v_{i}}\left(x\right)v_{i}|\sum_{i=1}^{3}\theta_{v_{i}}\left(x\right)v_{i}\right)=\left(\sum_{i=1}^{2}\ell_{v_{i}}\left(x\right)v_{i}+\left(\ell_{v_{3}}\left(x\right)-x^{k}\iota_{v_{3}}\left(x\right)\right)v_{3}|\sum_{i=1}^{3}\theta_{v_{i}}\left(x\right)v_{i}\right)+v_{3}x^{k}\left(\sum_{i=1}^{3}\iota_{v_{i}}\left(x\right)v_{i}|0\right).$$

Thus, $\left(\sum_{i=1}^{3}\ell_{v_{i}}\left(x\right)v_{i}|\sum_{i=1}^{3}\theta_{v_{i}}\left(x\right)v_{i}\right)\subset D$, showing that C⊂D. Consequently, D=C. Thus, we arrive at $\deg\left(\ell_{v_{3}}\left(x\right)\right)<\deg\left(\iota_{v_{3}}\left(x\right)\right)$ by reducing $\deg(\ell_{v_{3}}\left(x\right))$. □

**Lemma 4.**  *Let C be a double cyclic code with length (m,n) over R, and set*

$$C=\langle (\iota (x)|0),(\ell (x)|\theta (x))\rangle =\langle \left(\sum_{i=1}^{3}\iota_{v_{i}}\left(x\right)v_{i}|0\right),\left(\sum_{i=1}^{3}\ell_{v_{i}}\left(x\right)v_{i}|\sum_{i=1}^{3}\theta_{v_{i}}\left(x\right)v_{i}\right)\rangle .$$

*Then, $\iota_{v_{i}}\left(x\right)|\frac{x^{n}-1}{\theta_{v_{i}}\left(x\right)}\ell_{v_{i}}\left(x\right)$ for i=1,2,3.*

**Proof.**  By the proof of Proposition 2, we know that $\mathrm{Ker}\left(P_{n}{|}_{C}\right)=\langle (\iota (x)|0)\rangle$, where $P_{n}{|}_{C}$ is the second coordinate projection restricted to *C*. We concern about the codeword to $\frac{x^{n}-1}{\theta (x)}\left(\ell \left(x\right)|\theta \left(x\right)\right)$.

Since $\frac{x^{n}-1}{\theta (x)}\left(\ell \left(x\right)|\theta \left(x\right)\right)=\left(\frac{x^{n}-1}{\theta (x)}\ell \left(x\right)|0\right)\in \mathrm{Ker}\left(P_{n}{|}_{C}\right)$, we have $\iota \left(x\right)|\frac{x^{n}-1}{\theta (x)}\ell \left(x\right)$. From Lemma 1, we obtain $\iota_{v_{i}}\left(x\right)|\frac{x^{n}-1}{\theta_{v_{i}}\left(x\right)}\ell_{v_{i}}\left(x\right)$ for i=1,2,3. □

**Lemma 5.**  *Let C be a double cyclic code with length (m,n) over R, and set*

$$C=\langle (\iota (x)|0),(\ell (x)|\theta (x))\rangle =\langle \left(\sum_{i=1}^{3}\iota_{v_{i}}\left(x\right)v_{i}|0\right),\left(\sum_{i=1}^{3}\ell_{v_{i}}\left(x\right)v_{i}|\sum_{i=1}^{3}\theta_{v_{i}}\left(x\right)v_{i}\right)\rangle .$$

*Then, $\iota_{v_{i}}\left(x\right)|\frac{x^{n}-1}{\theta_{v_{i}}\left(x\right)}\gcd\left(\iota_{v_{i}}\left(x\right),\ell_{v_{i}}\left(x\right)\right)$ for i=1,2,3.*

**Proof.**  From Proposition 2, we have $\theta_{v_{i}}\left(x\right)|\left(x^{n}-1\right)$ for i=1,2,3. This implies that $\iota_{v_{i}}\left(x\right)|\frac{x^{n}-1}{\theta_{v_{i}}\left(x\right)}\iota_{v_{i}}\left(x\right)$ for i=1,2,3. By Lemma 4, we have $\iota_{v_{i}}\left(x\right)|\frac{x^{n}-1}{\theta_{v_{i}}\left(x\right)}\ell_{v_{i}}\left(x\right)$ for i=1,2,3. Therefore,

$$\iota_{v_{i}}\left(x\right)|\gcd\left(\frac{x^{n}-1}{\theta_{v_{i}}\left(x\right)}\iota_{v_{i}}\left(x\right),\frac{x^{n}-1}{\theta_{v_{i}}\left(x\right)}\ell_{v_{i}}\left(x\right)\right)|\frac{x^{n}-1}{\theta_{v_{i}}\left(x\right)}\gcd\left(\iota_{v_{i}}\left(x\right),\ell_{v_{i}}\left(x\right)\right),\;\mathrm{for}\;i=1,2,3.$$

Consequently, $\iota_{v_{i}}\left(x\right)|\frac{x^{n}-1}{\theta_{v_{i}}\left(x\right)}\gcd\left(\iota_{v_{i}}\left(x\right),\ell_{v_{i}}\left(x\right)\right)\;\mathrm{for}\;i=1,2,3$. □

**Definition 2.**  *Given C in the form of a double cyclic code with length (m,n) over R, a separable double cyclic code is one in which C is the direct product of $C_{m}$ and $C_{n}$.*

**Lemma 6.**  *If $C=\langle \left(\sum_{i=1}^{3}\iota_{v_{i}}\left(x\right)v_{i}|0\right),\left(\sum_{i=1}^{3}\ell_{v_{i}}\left(x\right)v_{i}|\sum_{i=1}^{3}\theta_{v_{i}}\left(x\right)v_{i}\right)\rangle$ is a separable R-double cyclic code, then $\ell_{v_{i}}\left(x\right)=0$ for i=1,2,3.*

**Proof.**  Consider the generator polynomial $\left(\ell \left(x\right)|\theta \left(x\right)\right)=\left(\sum_{i=1}^{3}\ell_{v_{i}}\left(x\right)v_{i}|\sum_{i=1}^{3}\theta_{v_{i}}\left(x\right)v_{i}\right)$. Since *C* a separable *R*-double cyclic code, it means that $\ell \left(x\right)=\sum_{i=1}^{3}\ell_{v_{i}}\left(x\right)v_{i}$ is the polynomial corresponding to a codeword of a cyclic code of length *m* over *R*. From the proof of Proposition 2, we know that the generator polynomial of the cyclic code of the first coordinate projection of all vectors in *C* is $\iota \left(x\right)=\sum_{i=1}^{3}\iota_{v_{i}}\left(x\right)v_{i}$. This means that

$$\sum_{i=1}^{3}\ell_{v_{i}}\left(x\right)v_{i}=\ell \left(x\right)\in \langle (\iota (x))\rangle =\langle (\sum_{i=1}^{3}\iota_{v_{i}}\left(x\right)v_{i})\rangle .$$

Then, $\sum_{i=1}^{3}\iota_{v_{i}}\left(x\right)v_{i}=\iota \left(x\right)|\ell \left(x\right)=\sum_{i=1}^{3}\ell_{v_{i}}\left(x\right)v_{i}$. Therefore, $\iota_{v_{i}}\left(x\right)|\ell_{v_{i}}\left(x\right),i=1,2,3$. But by Lemma 3, we have $\deg\left(\ell_{v_{i}}\left(x\right)\right)<\deg\left(\iota_{v_{i}}\left(x\right)\right)$ for i=1,2,3. This forces the conclusion to hold. □

Combining Propositions 1 and 2, and Lemmas 3, 4, 5, and 6 in this section, we obtain the first significant theorem of this paper.

**Theorem 1.**  *Let C be an R-double cyclic code of length (m,n), and let $C,C^{⊥}$ have the forms:*

$$\left\{\begin{array}{cc} C & =\langle (\iota (x)|0),(\ell (x)|\theta (x))\rangle =\langle \left(\sum_{i=1}^{3}\iota_{v_{i}}\left(x\right)v_{i}|0\right),\left(\sum_{i=1}^{3}\ell_{v_{i}}\left(x\right)v_{i}|\sum_{i=1}^{3}\theta_{v_{i}}\left(x\right)v_{i}\right)\rangle ,\\ C^{⊥} & =\langle \left(\bar{\iota }\left(x\right)|0\right),\left(\bar{\ell }\left(x\right)|\bar{\theta }\left(x\right)\right)\rangle =\langle \left(\sum_{i=1}^{3}{\bar{\iota }}_{v_{i}}\left(x\right)v_{i}|0\right),\left(\sum_{i=1}^{3}{\bar{\ell }}_{v_{i}}\left(x\right)v_{i}|\sum_{i=1}^{3}{\bar{\theta }}_{v_{i}}\left(x\right)v_{i}\right)\rangle . \end{array}\right.$$

*Then, $\iota_{v_{i}}\left(x\right)|x^{m}-1$ and $\theta_{v_{i}}\left(x\right)|x^{n}-1$ for i=1,2,3.*

*If C is a separable R-double cyclic code, then $\ell_{v_{i}}\left(x\right)=0$ for i=1,2,3.*

*If C is a free R-double cyclic code, then the following statements hold:*

*(1) $\deg\left(\ell_{v_{i}}\left(x\right)\right)<\deg\left(\iota_{v_{i}}\left(x\right)\right)$ for i=1,2,3.*

*(2) $\iota_{v_{i}}\left(x\right)|\frac{x^{n}-1}{\theta_{v_{i}}\left(x\right)}\gcd\left(\iota_{v_{i}}\left(x\right),\ell_{v_{i}}\left(x\right)\right)$ for i=1,2,3.*

## 4. Generator Matrices

In this section, we consider the generating matrices of *R*-double cyclic codes.

**Proposition 3.**  *Let $C=\langle (\iota (x)|0),(\ell (x)|\theta (x))\rangle =\langle \left(\sum_{i=1}^{3}\iota_{v_{i}}\left(x\right)v_{i}|0\right),\left(\sum_{i=1}^{3}\ell_{v_{i}}\left(x\right)v_{i}|\sum_{i=1}^{3}\theta_{v_{i}}\left(x\right)v_{i}\right)\rangle$ be a double cyclic code with length (m,n) over R. Then, C is permutation equal to a $F_{q}$-linear code with a generator matrix in the form $G=\left(\begin{array}{c} G_{1}v_{1}\\ G_{2}v_{2}\\ G_{3}v_{3} \end{array}\right)$, where*

$$G_{i}=\left(\begin{array}{c} \begin{array}{ccc} I_{m-\deg(\iota_{v_{i}}\left(x\right))} & A_{i}^{1} & A_{i}^{2}\\ 0 & B_{i}^{1} & B_{i}^{2}\\ 0 & 0 & 0 \end{array} \end{array}|\begin{array}{ccc} 0 & 0 & 0\\ B_{i}^{3} & I_{k_{i}} & 0\\ M_{i}^{1} & M_{i}^{2} & I_{n-\deg\left(\iota_{v_{i}}\left(x\right)\right)-k_{i}} \end{array}\right),i=1,2,3$$

*and $k_{i}=\deg\left(\iota_{v_{i}}\left(x\right)\right)-\deg\left(\gcd\left(\iota_{v_{i}}\left(x\right),\ell_{v_{i}}\left(x\right)\right)\right)\in N$, i=1,2,3.*

**Proof.**  We know that all of $C_{v_{i}},i=1,2,3$ are double cyclic codes over $F_{q}$ from the canonical projections. By Proposition 8 of paper [7], we know that each of $C_{v_{i}}$ is a permutation equivalent to a linear code over $F_{q}$, and their generator matrices have the following forms:

$$G_{i}=\left(\begin{array}{c} \begin{array}{ccc} I_{m-\deg(\iota_{v_{i}}\left(x\right))} & A_{i}^{1} & A_{i}^{2}\\ 0 & B_{i}^{1} & B_{i}^{2}\\ 0 & 0 & 0 \end{array} \end{array}|\begin{array}{ccc} 0 & 0 & 0\\ B_{i}^{3} & I_{k_{i}} & 0\\ M_{i}^{1} & M_{i}^{2} & I_{n-\deg\left(\iota_{v_{i}}\left(x\right)\right)-k_{i}} \end{array}\right),i=1,2,3,$$

where $B_{i}^{1}$ are full rank square matrices of size $k_{i}\times k_{i}$, i=1,2,3. By reducing the canonical projections to the double cyclic codes, we obtain that the matrix form of *C* is *G*. □

From the generator matrix of the *R*-double cyclic codes, we have an easy computation, as follows.

**Corollary 1.**  *Let $C=\langle (\iota (x)|0),(\ell (x)|\theta (x))\rangle =\langle \left(\sum_{i=1}^{3}\iota_{v_{i}}\left(x\right)v_{i}|0\right),\left(\sum_{i=1}^{3}\ell_{v_{i}}\left(x\right)v_{i}|\sum_{i=1}^{3}\theta_{v_{i}}\left(x\right)v_{i}\right)\rangle$ be a double cyclic code with length (m,n) over R. Then, C is an $F_{q}$-linear code of dimension $3m+3n-\sum_{i=1}^{3}\left(\deg\left(\iota_{v_{i}}\left(x\right)\right)+\deg\left(\theta_{v_{i}}\left(x\right)\right)\right)$.*

**Proposition 4.**  *Let $C=\langle (\iota (x)|0),(\ell (x)|\theta (x))\rangle =\langle \left(\sum_{i=1}^{3}\iota_{v_{i}}\left(x\right)v_{i}|0\right),\left(\sum_{i=1}^{3}\ell_{v_{i}}\left(x\right)v_{i}|\sum_{i=1}^{3}\theta_{v_{i}}\left(x\right)v_{i}\right)\rangle$ be a double cyclic code with length (m,n) over R, and*

$$C^{⊥}=\langle \left(\bar{\iota }\left(x\right)|0\right),\left(\bar{\ell }\left(x\right)|\bar{\theta }\left(x\right)\right)\rangle =\langle \left(\sum_{i=1}^{3}{\bar{\iota }}_{v_{i}}\left(x\right)v_{i}|0\right),\left(\sum_{i=1}^{3}{\bar{\ell }}_{v_{i}}\left(x\right)v_{i}|\sum_{i=1}^{3}{\bar{\theta }}_{v_{i}}\left(x\right)v_{i}\right)\rangle .$$

*Then,*

$$\left\{\begin{array}{cc} |C_{m}|=q^{3m+\sum_{i=1}^{3}k_{i}-\sum_{i=1}^{3}\deg\left(\iota_{v_{i}}\left(x\right)\right)}, & |C_{n}|=q^{3n-\sum_{i=1}^{3}\deg\left(\theta_{v_{i}}\left(x\right)\right)},\\ |{\left(C^{⊥}\right)}_{m}|=q^{\sum_{i=1}^{3}\deg\left(\theta_{v_{i}}\left(x\right)\right)}, & |{\left(C^{⊥}\right)}_{n}|=q^{\sum_{i=1}^{3}\deg\left(\theta_{v_{i}}\left(x\right)\right)+\sum_{i=1}^{3}k_{i}}, \end{array}\right.$$

*where $k_{i}=\deg\left(\iota_{v_{i}}\left(x\right)\right)-\deg\left(\gcd\left(\iota_{v_{i}}\left(x\right),\ell_{v_{i}}\left(x\right)\right)\right)\in N$, i=1,2,3.*

**Proof.**  We know that $C_{m}$ is generated by the polynomial gcd(ι(x),ℓ(x)), and $C_{n}$ is generated by θ(x), in accordance with Proposition 2. By mapping the codewords to single cyclic codes over *R*, we obtain that

$$\left\{\begin{array}{c} |C_{m}|=\sum_{i=1}^{3}\left|{\left(C_{m}\right)}_{v_{i}}\right|=\sum_{i=1}^{3}q^{m-\deg(\iota_{v_{i}}\left(x\right),\ell_{v_{i}}\left(x\right))}=q^{3m+\sum_{i=1}^{3}k_{i}-\sum_{i=1}^{3}\deg\left(\iota_{v_{i}}\left(x\right)\right)};\\ |C_{n}|=\sum_{i=1}^{3}\left|{\left(C_{n}\right)}_{v_{i}}\right|=\sum_{i=1}^{3}q^{n-\deg(\theta_{v_{i}}\left(x\right))}=q^{3n-\sum_{i=1}^{3}\deg\left(\theta_{v_{i}}\left(x\right)\right)}. \end{array}\right.$$

From the generating matrix forms of *C*, we can efficiently work out the parity check matrix of *C* is $H=\left(\begin{array}{c} H_{1}v_{1}\\ H_{2}v_{2}\\ H_{3}v_{3} \end{array}\right)$, where

$$H_{i}=\left(\begin{array}{cc} \begin{array}{ccc} {\left(A_{i}^{1}\right)}^{t} & I_{k_{i}} & 0\\ {\left(A_{i}^{2}\right)}^{t} & 0 & I_{\deg\left(\iota_{v_{i}}\left(x\right)\right)-k_{i}}\\ 0 & 0 & 0 \end{array}| & \begin{array}{ccc} 0 & {\left(B_{i}^{1}\right)}^{t} & {\left(B_{i}^{1}\right)}^{t}{\left(M_{i}^{2}\right)}^{t}\\ 0 & {\left(B_{i}^{2}\right)}^{t} & {\left(B_{i}^{2}\right)}^{t}{\left(M_{i}^{2}\right)}^{t}\\ I_{\deg(\theta_{v_{i}}\left(x\right))} & {\left(B_{i}^{3}\right)}^{t} & {\left(M_{i}^{1}\right)}^{t}+{\left(B_{i}^{3}\right)}^{t}{\left(M_{i}^{2}\right)}^{t} \end{array} \end{array}\right),i=1,2,3.$$

Taking advantage of the connection between cyclic codes and their dual codes about the matrix forms, we can apply the same approach as before to obtain that

$$\left\{\begin{array}{c} |{\left(C^{⊥}\right)}_{m}|=\sum_{i=1}^{3}\left|{\left({\left(C^{⊥}\right)}_{m}\right)}_{v_{i}}\right|=q^{\sum_{i=1}^{3}\deg\left(\theta_{v_{i}}\left(x\right)\right)},\\ |{\left(C^{⊥}\right)}_{n}|=\sum_{i=1}^{3}\left|{\left({\left(C^{⊥}\right)}_{n}\right)}_{v_{i}}\right|=q^{\sum_{i=1}^{3}\deg\left(\theta_{v_{i}}\left(x\right)\right)+\sum_{i=1}^{3}k_{i}}. \end{array}\right.$$

□

**Corollary 2.**  *Let C and $C^{⊥}$ be defined as presented above. Then,*

$$\left\{\begin{array}{c} \deg\left({\bar{\iota }}_{v_{i}}\left(x\right)\right)=m-\deg\left(\gcd\left(\iota_{v_{i}}\left(x\right),\ell_{v_{i}}\left(x\right)\right)\right),\\ \deg\left({\bar{\theta }}_{v_{i}}\left(x\right)\right)=n-\deg\left(\iota_{v_{i}}\left(x\right)\right)-\deg\left(\theta_{v_{i}}\left(x\right)\right)+\deg\left(\gcd\left(\iota_{v_{i}}\left(x\right),\ell_{v_{i}}\left(x\right)\right)\right), \end{array}\right.i=1,2,3.$$

**Proof.**  Given that ${\left(C_{m}\right)}^{⊥}$ is a cyclic code generated by $\bar{\iota }\left(x\right)$, we can deduce from the findings on cyclic codes over *R* that $|{\left({\left(C_{m}\right)}^{⊥}\right)}_{v_{i}}|=q^{m-\deg(\iota_{v_{i}}\left(x\right))}$ for i=1,2,3. Furthermore, by Proposition 4, we have $|{\left({\left(C_{m}\right)}^{⊥}\right)}_{v_{i}}|=q^{\deg\left(\iota_{v_{i}}\left(x\right)\right)-k_{i}},i=1,2,3$. Thus, $\deg\left({\bar{\iota }}_{v_{i}}\left(x\right)\right)=m-\deg\left(\gcd\left(\iota_{v_{i}}\left(x\right),\ell_{v_{i}}\left(x\right)\right)\right),i=1,2,3$.

Because ${\left(C^{⊥}\right)}_{m}$ is a cyclic code generated by $\bar{\theta }\left(x\right)$, and $C^{⊥}$ is also a *R*-double cyclic code of length (m,n), therefore, $|{\left({\left(C^{⊥}\right)}_{m}\right)}_{v_{i}}|=q^{n-\deg({\bar{\theta }}_{v_{i}}\left(x\right))}$. According to Proposition 4, we have $|{\left({\left(C^{⊥}\right)}_{m}\right)}_{v_{i}}|=q^{\deg\left(\theta_{v_{i}}\left(x\right)\right)+k_{i}}$ for i=1,2,3. Consequently,

$$\deg\left({\bar{\theta }}_{v_{i}}\left(x\right)\right)=n-\deg\left(\iota_{v_{i}}\left(x\right)\right)-\deg\left(\theta_{v_{i}}\left(x\right)\right)+\deg\left(\gcd\left(\iota_{v_{i}}\left(x\right),\ell_{v_{i}}\left(x\right)\right)\right),i=1,2,3.$$

□

## 5. Dual Codes over $F_{q}+vF_{q}+v^{2}F_{q}$

In this section, we mainly consider the generating polynomials of dual codes of double cyclic codes over $F_{q}+vF_{q}+v^{2}F_{q}$.

**Definition 3.**  *Let $r\left(x\right)=\sum_{i=1}^{3}r_{v_{i}}\left(x\right)v_{i}$ with $r_{v_{i}}\left(x\right)\in F_{q}\left[x\right],i=1,2,3$ for r(x)∈R[x]. Provide an explanation of the monic reciprocal polynomial r(x), which is defined as*

$$r^{*}\left(x\right)=\sum_{i=1}^{3}r_{v_{i}}^{*}\left(x\right)v_{i}=\sum_{i=1}^{3}{\left(lc\left(r_{v_{i}}\left(x\right)\right)\right)}^{-1}x^{\deg(r_{v_{i}}\left(x\right))}r_{v_{i}}\left(x^{-1}\right)v_{i},$$

*where $lc(r_{v_{i}}\left(x\right))$ is expressed by the lowest term coefficient of $r_{v_{i}}\left(x\right)$ for i=1,2,3.*

**Remark 3.**  The definition of the monic reciprocal polynomial in R[x] can be regarded as a generalization to the case of finite fields. According to this definition, we also have ${\left(r^{*}\left(x\right)\right)}_{v_{i}}={\left(r_{v_{i}}\left(x\right)\right)}^{*},i=1,2,3$. For this reason, we can write $r_{v_{i}}^{*}\left(x\right)$, i=1,2,3 without confusion.

As in Lemma 1, we also have the following.

**Lemma 7.**  Let a(x),b(x)∈R[x] with a(x)|b(x). Then, ${\left(\frac{b(x)}{a(x)}\right)}^{*}=\frac{b^{*}\left(x\right)}{a^{*}\left(x\right)}$.

**Proof.**  It is equivalent to prove ${\left(a\left(x\right)b\left(x\right)\right)}^{*}=a^{*}\left(x\right)b^{*}\left(x\right)$ for a(x),b(x)∈R[x]. We obtain ${\left(f\left(x\right)g\left(x\right)\right)}^{*}=f^{*}\left(x\right)g^{*}\left(x\right)$, $f\left(x\right),g\left(x\right)\in F_{q}\left[x\right]$ by applying polynomial theory to conventional finite fields, while we can combine $\left\{v_{1},v_{2},v_{3}\right\}$ to decompose r(x)inR[x], which corresponds to the case of the polynomial over finite fields. Then, we obtain the results in this way. □

**Remark 4.**  We still have $r^{**}\left(x\right)=r\left(x\right),\forall r\left(x\right)\in R\left[x\right]$, as in the case of finite fields. In the next decomposition of polynomials, we will repeatedly use this Lemma, as well as Lemmas 1 and 2, without further explanation.

**Proposition 5.**  *Using the findings from Lemma 6, let*

$$C=\langle (\iota (x)|0),(0|\theta (x))\rangle =\langle \left(\sum_{i=1}^{3}\iota_{v_{i}}\left(x\right)v_{i}|0\right),\left(\sum_{i=1}^{3}\ell_{v_{i}}\left(x\right)v_{i}|\sum_{i=1}^{3}\theta_{v_{i}}\left(x\right)v_{i}\right)\rangle$$

*be a separable R-double cyclic code of length (m,n). Additionally, $C^{⊥}$ is a separable R-double cyclic code, and*

$$C^{⊥}=\langle \left(\frac{x^{m}-1}{\iota^{*}\left(x\right)}|0\right),\left(0|\frac{x^{n}-1}{\theta^{*}\left(x\right)}\right)\rangle =\langle \left(\sum_{i=1}^{3}\frac{x^{m}-1}{\iota_{v_{i}}^{*}\left(x\right)}v_{i}|0\right),\left(0|\sum_{i=1}^{3}\frac{x^{n}-1}{\theta_{v_{i}}^{*}\left(x\right)}v_{i}\right)\rangle .$$

**Proof.**  Since *C* is separable, we have $C=C_{m}\times C_{n}$. Thus, it is easy to obtain $C^{⊥}=C_{m}^{⊥}\times C_{n}^{⊥}$. Referring to cyclic codes over $F_{q}+vF_{q}+v^{2}F_{q}$ in [19], we achieve that

$$C^{⊥}=\langle \left(\frac{x^{m}-1}{\iota^{*}\left(x\right)}|0\right),\left(0|\frac{x^{n}-1}{\theta^{*}\left(x\right)}\right)\rangle =\langle \left(\sum_{i=1}^{3}\frac{x^{m}-1}{\iota_{v_{i}}^{*}\left(x\right)}v_{i}|0\right),\left(0|\sum_{i=1}^{3}\frac{x^{n}-1}{\theta_{v_{i}}^{*}\left(x\right)}v_{i}\right)\rangle .$$

□

Let $\omega_{m}\left(x\right)$ represent the polynomial $\sum_{i=0}^{m-1}x^{i}$. Using this symbol, we can easily justify that

**Lemma 8.**  *Let m,n∈N; then, $x^{mn}-1=\left(x^{m}-1\right)\omega_{n}\left(x^{m}\right)$.*

**Definition 4.**  *Let $c\left(x\right)=\left(\sum_{i=1}^{3}c_{v_{i}}^{1}\left(x\right)v_{i}|\sum_{i=1}^{3}c_{v_{i}}^{2}\left(x\right)v_{i}\right),d\left(x\right)=\left(\sum_{i=1}^{3}d_{v_{i}}^{1}\left(x\right)v_{i}|\sum_{i=1}^{3}d_{v_{i}}^{2}\left(x\right)v_{i}\right)$ be two elements in $R_{m,n}\left[x\right]$. We define the map $\circ :R_{m,n}\left[x\right]\times R_{m,n}\left[x\right]\to R_{l}\left[x\right]$ as*

$$\circ \left(c\left(x\right),d\left(x\right)\right)=\sum_{i=1}^{3}\left(c_{v_{i}}^{1}\left(x\right)\omega_{\frac{l}{m}}\left(x^{m}\right)x^{l-1-\deg(d_{v_{i}}^{1}\left(x\right))}d_{v_{i}}^{1*}\left(x\right)+c_{v_{i}}^{2}\left(x\right)\theta_{\frac{l}{m}}\left(x^{m}\right)x^{l-1-\deg(d_{v_{i}}^{2}\left(x\right))}d_{v_{i}}^{2*}\left(x\right)\right)v_{i},$$

*where the right side of the equality is the combination of polynomials module $x^{l}-1$.*

We denote ∘(c(x),d(x)) by c(x)∘d(x) for the sake of simplicity and convenience.

**Lemma 9.**  *Let c,d be two vectors in $R^{m}\times R^{n}$, with corresponding polynomials*

$$c\left(x\right)=\left(\sum_{i=1}^{3}c_{v_{i}}^{1}\left(x\right)v_{i}|\sum_{i=1}^{3}c_{v_{i}}^{2}\left(x\right)v_{i}\right),d\left(x\right)=\left(\sum_{i=1}^{3}d_{v_{i}}^{1}\left(x\right)v_{i}|\sum_{i=1}^{3}d_{v_{i}}^{2}\left(x\right)v_{i}\right)$$

*respectively. Then, c is orthogonal to d and all of its shift, if and only if c(x)∘d(x)≡0.*

**Proof.**  Let $d_{\left(s\right)}=\left(d_{0+s}^{1},\cdots ,d_{m-1+s}^{1}|d_{0+s}^{2},\cdots ,d_{n-1+s}^{2}\right)$ be the *s*-th cyclic shift of vector *d*, 0≤s≤l−1. We know that $\langle c,d_{\left(s\right)}\rangle =0$, if and only if $\sum_{k_{1}=0}^{m-1}c_{k_{1}}^{1}d_{k_{1}+s}^{1}+\sum_{k_{2}=0}^{n-1}c_{k_{2}}^{2}d_{k_{2}+s}^{2}=0$. Fixing $\Delta_{s}=\sum_{k_{1}=0}^{m-1}c_{k_{1}}^{1}d_{k_{1}+s}^{1}+\sum_{k_{2}=0}^{n-1}c_{k_{2}}^{2}d_{k_{2}+s}^{2}$, we can obtain that

(1) $$\begin{array}{cc} c(x)\circ d(x) & =\sum_{i=0}^{m-1}\left(\omega_{\frac{l}{m}}\left(x^{m}\right)\sum_{k_{1}=0}^{m-1}c_{k_{1}}^{1}d_{k_{1}+i}^{1}x^{l-1-i}\right)+\sum_{j=0}^{n-1}\left(\omega_{\frac{l}{n}}\left(x^{n}\right)\sum_{k_{2}=0}^{n-1}c_{k_{2}}^{2}d_{k_{2}+j}^{2}x^{l-1-j}\right)\\ \; & =\omega_{\frac{l}{m}}\left(x^{m}\right)\left[\sum_{i=0}^{m-1}\sum_{k_{1}=0}^{m-1}c_{k_{1}}^{1}d_{k_{1}+i}^{1}x^{l-1-i}\right]+\omega_{\frac{l}{n}}\left(x^{n}\right)\left[\sum_{j=0}^{m-1}\sum_{k_{2}=0}^{n-1}c_{k_{2}}^{2}d_{k_{2}+j}^{2}x^{l-1-j}\right]\\ \; & =\sum_{s=0}^{l-1}\Delta_{s}x^{l-1-s} \end{array}$$

in $R\left[x\right]/\left(x^{l}-1\right)$. Hence, c(x)∘d(x)=0 only if and when $\Delta_{s}=0$ for all 0≤s≤l−1. □

**Lemma 10.**  Let $c\left(x\right)=(c^{1}\left(x\right)|c^{2}\left(x\right))$ and $d\left(x\right)=(d^{1}\left(x\right)|d^{2}\left(x\right))$ be two elements in the set $R_{m,n}\left[x\right]$, such that c(x)∘d(x)=0 mod $\left(x^{l}-1\right)$. When $c^{1}\left(x\right)\equiv 0$ or $d^{1}\left(x\right)\equiv 0$, then $c^{2}\left(x\right)d^{2*}\left(x\right)=0$ mod $\left(x^{n}-1\right)$. In turn, if $c^{2}\left(x\right)\equiv 0$ or $d^{2}\left(x\right)\equiv 0$, then $c^{1}\left(x\right)d^{1*}\left(x\right)=0$ mod $\left(x^{m}-1\right)$.

**Proof.**  Let $c^{2}\left(x\right)$ or $d^{2}\left(x\right)$ be equal to 0 module $x^{n}-1$. This means that $c_{v_{i}}^{2}\left(x\right)\equiv 0$, i=1,2,3 or $d_{v_{i}}^{2}\left(x\right)\equiv 0$, i=1,2,3. By the concrete definition forms of ∘ in R[x], we have

$$c\left(x\right)\circ d\left(x\right)=\sum_{i=1}^{3}\left(c_{v_{i}}^{1}\left(x\right)\omega_{\frac{l}{m}}\left(x^{m}\right)x^{l-1-\deg(d_{v_{i}}^{1}\left(x\right))}d_{v_{i}}^{1*}\left(x\right)\right)v_{i}=0\;\mathrm{mod}\;\left(x^{l}-1\right).$$

Therefore, a polynomial π(x)∈R[x] with $\pi \left(x\right)=\sum_{i=1}^{3}\pi_{v_{i}}\left(x\right)v_{i}$, such that

$$\sum_{i=1}^{3}\left(c_{v_{i}}^{1}\left(x\right)\omega_{\frac{l}{m}}\left(x^{m}\right)x^{l-1-\deg(d_{v_{i}}^{1}\left(x\right))}d_{v_{i}}^{1*}\left(x\right)\right)v_{i}=\pi \left(x\right)\left(x^{l}-1\right)=\sum_{i=1}^{3}\pi_{v_{i}}\left(x\right)\left(x^{l}-1\right)v_{i}.$$

exists.

Then, $c_{v_{i}}^{1}\left(x\right)\omega_{\frac{l}{m}}\left(x^{m}\right)x^{l-1-\deg(d_{v_{i}}^{1}\left(x\right))}d_{v_{i}}^{1*}\left(x\right)=\pi_{v_{i}}\left(x\right)\left(x^{l}-1\right),i=1,2,3$. Let $\Pi \left(x\right)=\sum_{i=1}^{3}\Pi_{v_{i}}\left(x\right)v_{i}$ with $\Pi_{v_{i}}\left(x\right)=x^{\deg(d_{v_{i}}^{1}\left(x\right))+1}\pi_{v_{i}}\left(x\right),i=1,2,3$; we have $c_{v_{i}}^{1}\left(x\right)\omega_{\frac{l}{m}}\left(x^{m}\right)x^{l}d_{v_{i}}^{1*}\left(x\right)=\Pi_{v_{i}}\left(x\right)\left(x^{l}-1\right),i=1,2,3$. By Lemma 8, we obtain $x^{l}-1=\omega_{\frac{l}{m}}\left(x^{m}\right)\left(x^{m}-1\right)$. So, $c_{v_{i}}^{1}\left(x\right)d_{v_{i}}^{1*}\left(x\right)x^{l}=\Pi_{v_{i}}\left(x\right)\left(x^{m}-1\right)\;i=1,2,3$. This means that $x^{m}-1|c_{v_{i}}^{1}\left(x\right)d_{v_{i}}^{1*}\left(x\right)x^{l},i=1,2,3$. Obviously, $x^{m}-1$ and $x^{l}$ are co-prime to each other. This leads to $x^{m}-1|c_{v_{i}}^{1}\left(x\right)d_{v_{i}}^{1*}\left(x\right),i=1,2,3$. Hence,

$$\sum_{i=1}^{3}c_{v_{i}}^{1}\left(x\right)d_{v_{i}}^{1*}\left(x\right)v_{i}=c^{1}\left(x\right)d^{1*}\left(x\right)=0\;\mathrm{mod}\;\left(x^{m}-1\right).$$

The same assertion can be proved for the other cases. □

**Proposition 6.**  *Let $C=\langle (\iota (x)|0),(\ell (x)|\theta (x))\rangle =\langle \left(\sum_{i=1}^{3}\iota_{v_{i}}\left(x\right)v_{i}|0\right),\left(\sum_{i=1}^{3}\ell_{v_{i}}\left(x\right)v_{i}|\sum_{i=1}^{3}\theta_{v_{i}}\left(x\right)v_{i}\right)\rangle$ be a double cyclic code of length (m,n) over R and its dual,*

$$C^{⊥}=\langle \left(\bar{\iota }\left(x\right)|0\right),\left(\bar{\ell }\left(x\right)|\bar{\theta }\left(x\right)\right)\rangle =\langle \left(\sum_{i=1}^{3}{\bar{\iota }}_{v_{i}}\left(x\right)v_{i}|0\right),\left(\sum_{i=1}^{3}{\bar{\ell }}_{v_{i}}\left(x\right)v_{i}|\sum_{i=1}^{3}{\bar{\theta }}_{v_{i}}\left(x\right)v_{i}\right)\rangle .$$

Then, $\bar{\iota }\left(x\right)=\frac{x^{m}-1}{\gcd^{*}\left(\iota \left(x\right),\ell \left(x\right)\right)}=\sum_{i=1}^{3}\frac{x^{m}-1}{\gcd^{*}\left(\iota_{v_{i}}\left(x\right),\ell_{v_{i}}\left(x\right)\right)}v_{i}$.

**Proof.**  Obviously, $\left(\bar{\iota }\left(x\right)|0\right)$ belongs to $C^{⊥}$. As a consequence of Lemma 9, we have

$$\left\{\begin{array}{cc} \left(\bar{\iota }\left(x\right)|0\right)\circ \left(\iota \left(x\right)|0\right) & =0\;\mathrm{mod}\;(x^{l}-1),\\ \left(\bar{\iota }\left(x\right)|0\right)\circ \left(\ell \left(x\right)|\theta \left(x\right)\right) & =0\;\mathrm{mod}\;(x^{l}-1). \end{array}\right.$$

Therefore, by Lemma 10, we also have

$$\left\{\begin{array}{cc} {\bar{\iota }}^{*}\left(x\right)\iota \left(x\right) & =0\;\mathrm{mod}\;\left(x^{m}-1\right)\Leftrightarrow \left(x^{m}-1\right)|{\bar{\iota }}^{*}\left(x\right)\iota \left(x\right),\\ {\bar{\iota }}^{*}\left(x\right)\ell \left(x\right) & =0\;\mathrm{mod}\;\left(x^{m}-1\right)\Leftrightarrow \left(x^{m}-1\right)|{\bar{\iota }}^{*}\left(x\right)\ell \left(x\right). \end{array}\right.$$

Then, we obtain $x^{m}-1\left|\gcd\left({\bar{\iota }}^{*}\left(x\right)\iota \left(x\right),{\bar{\iota }}^{*}\left(x\right)\ell \left(x\right)\right)\right|{\bar{\iota }}^{*}\left(x\right)\gcd\left(\iota \left(x\right),\ell \left(x\right)\right)$, while $x^{m}-1|{\bar{\iota }}^{*}\left(x\right)\gcd$(ι(x),ℓ(x)), if and only if

$$x^{m}-1|{\bar{\iota }}_{v_{i}}^{*}\left(x\right)\gcd_{v_{i}}\left(\iota \left(x\right),\ell \left(x\right)\right)={\bar{\iota }}_{v_{i}}^{*}\left(x\right)\gcd\left(\iota_{v_{i}}\left(x\right),\ell_{v_{i}}\left(x\right)\right),i=1,2,3.$$

Since all of ${\bar{\iota }}_{v_{i}}^{*}\left(x\right),\gcd\left(\iota_{v_{i}}\left(x\right),\ell_{v_{i}}\left(x\right)\right)$, i=1,2,3 are factors of $x^{m}-1$, by Corollary 2, we have

$$\deg\left({\bar{\iota }}_{v_{i}}^{*}\left(x\right)\right)=\deg\left({\bar{\iota }}_{v_{i}}\left(x\right)\right)=m-\deg\left(\gcd\left(\iota_{v_{i}}\left(x\right),\ell_{v_{i}}\left(x\right)\right)\right),i=1,2,3.$$

Therefore, $x^{m}-1={\bar{\iota }}_{v_{i}}^{*}\left(x\right)\gcd_{v_{i}}\left(\iota \left(x\right),\ell \left(x\right)\right)={\bar{\iota }}_{v_{i}}^{*}\left(x\right)\gcd\left(\iota_{v_{i}}\left(x\right),\ell_{v_{i}}\left(x\right)\right)$, i=1,2,3. Then, we obtain

$${\bar{\iota }}^{*}\left(x\right)\gcd\left(\iota \left(x\right),\ell \left(x\right)\right)=\sum_{i=1}^{3}{\bar{\iota }}_{v_{i}}^{*}\left(x\right)\gcd\left(\iota_{v_{i}}\left(x\right),\ell_{v_{i}}\left(x\right)\right)v_{i}=\sum_{i=1}^{3}\left(x^{m}-1\right)v_{i}=x^{m}-1.$$

Consequently, we have $\bar{\iota }\left(x\right)=\frac{x^{m}-1}{\gcd^{*}\left(\iota \left(x\right),\ell \left(x\right)\right)}=\sum_{i=1}^{3}\frac{x^{m}-1}{\gcd^{*}\left(\iota_{v_{i}}\left(x\right),\ell_{v_{i}}\left(x\right)\right)}v_{i}$. □

**Proposition 7.**  *Let $C=\langle (\iota (x)|0),(\ell (x)|\theta (x))\rangle =\langle \left(\sum_{i=1}^{3}\iota_{v_{i}}\left(x\right)v_{i}|0\right),\left(\sum_{i=1}^{3}\ell_{v_{i}}\left(x\right)v_{i}|\sum_{i=1}^{3}\theta_{v_{i}}\left(x\right)v_{i}\right)\rangle$ be an R-double cyclic code of length (m,n) and its dual,*

$$C^{⊥}=\langle \left(\bar{\iota }\left(x\right)|0\right),\left(\bar{\ell }\left(x\right)|\bar{\theta }\left(x\right)\right)\rangle =\langle \left(\sum_{i=1}^{3}{\bar{\iota }}_{v_{i}}\left(x\right)v_{i}|0\right),\left(\sum_{i=1}^{3}{\bar{\ell }}_{v_{i}}\left(x\right)v_{i}|\sum_{i=1}^{3}{\bar{\theta }}_{v_{i}}\left(x\right)v_{i}\right)\rangle .$$

Then, $\bar{\theta }\left(x\right)=\frac{\left(x^{n}-1\right)\gcd^{*}\left(\iota \left(x\right),\ell \left(x\right)\right)}{\iota^{*}\left(x\right)\theta^{*}\left(x\right)}=\sum_{i=1}^{3}\frac{\left(x^{n}-1\right)\gcd^{*}\left(\iota_{v_{i}}\left(x\right),\ell_{v_{i}}\left(x\right)\right)}{\iota_{v_{i}}^{*}\left(x\right)\theta_{v_{i}}^{*}\left(x\right)}v_{i}$.

**Proof.**  Considering the codeword

$$\left(0|\frac{\iota (x)}{\gcd(\iota (x),\ell (x))}\theta \left(x\right)\right)=\frac{\iota (x)}{\gcd(\iota (x),\ell (x))}\left(\ell \left(x\right)|\theta \left(x\right)\right)-\frac{\ell (x)}{\gcd(\iota (x),\ell (x))}\left(\iota \left(x\right)|0\right),$$

we have $\left(0|\frac{\iota (x)}{\gcd(\iota (x),\ell (x))}\theta \left(x\right)\right)\in C$. By Lemma 9, we obtain

$$\left(\bar{\ell }\left(x\right)|\bar{\theta }\left(x\right)\right)\circ \left(0|\frac{\iota (x)}{\gcd(\iota (x),\ell (x))}\theta \left(x\right)\right)=0\;\mathrm{mod}\;\left(x^{l}-1\right).$$

Then, from Lemma 10, we obtain that

$$\bar{\theta }\left(x\right)\frac{\iota^{*}\left(x\right)\theta^{*}\left(x\right)}{\gcd^{*}\left(\iota \left(x\right),\ell \left(x\right)\right)}=0\;\mathrm{mod}\;\left(x^{n}-1\right)\Leftrightarrow x^{n}-1|\bar{\theta }\left(x\right)\frac{\iota^{*}\left(x\right)\theta^{*}\left(x\right)}{\gcd^{*}\left(\iota \left(x\right),\ell \left(x\right)\right)}.$$

while $x^{n}-1|\bar{\theta }\left(x\right)\frac{\iota^{*}\left(x\right)\theta^{*}\left(x\right)}{\gcd*(\iota (x),\ell (x))}$, if and only if

$$x^{n}-1|{\bar{\theta }}_{v_{i}}\left(x\right)\frac{\iota_{v_{i}}^{*}\left(x\right)\theta_{v_{i}}^{*}\left(x\right)}{\gcd_{v_{i}}^{*}\left(\iota \left(x\right),\ell \left(x\right)\right)}={\bar{\theta }}_{v_{i}}\left(x\right)\frac{\iota_{v_{i}}^{*}\left(x\right)\theta_{v_{i}}^{*}\left(x\right)}{\gcd^{*}\left(\iota_{v_{i}}\left(x\right),\ell_{v_{i}}\left(x\right)\right)},i=1,2,3.$$

From Theorem 1, we acquire ${\bar{\theta }}_{v_{i}}\left(x\right)|\left(x^{n}-1\right),i=1,2,3$. Simultaneously, by Lemma 5, we have that $\frac{\iota_{v_{i}}^{*}\left(x\right)\theta_{v_{i}}^{*}\left(x\right)}{\gcd(\iota_{v_{i}}\left(x\right),\ell_{v_{i}}\left(x\right))}|\left(x^{n}-1\right),i=1,2,3$. From Corollary 2, we gain

$$\deg\left({\bar{\theta }}_{v_{i}}\left(x\right)\right)=n-\deg\left(\iota_{v_{i}}\left(x\right)\right)-\deg\left(\theta_{v_{i}}\left(x\right)\right)+\deg(\gcd\left(\xi_{v_{i}}\left(x\right),\ell_{v_{i}}\left(x\right)\right),i=1,2,3.$$

Therefore, $\deg(\frac{{\bar{\theta }}_{v_{i}}\left(x\right)\iota_{v_{i}}^{*}\left(x\right)\theta_{v_{i}}^{*}\left(x\right)}{\gcd^{*}\left(\iota_{v_{i}}\left(x\right),\ell_{v_{i}}\left(x\right)\right)})=n,i=1,2,3$. This indicates that $x^{n}-1=\frac{{\bar{\theta }}_{v_{i}}\left(x\right)\iota_{v_{i}}^{*}\left(x\right)\theta_{v_{i}}^{*}\left(x\right)}{\gcd^{*}\left(\iota_{v_{i}}\left(x\right),\ell_{v_{i}}\left(x\right)\right)},i=1,2,3$. Hence,

$$\bar{\theta }\left(x\right)\frac{\iota^{*}\left(x\right)\theta^{*}\left(x\right)}{\gcd^{*}\left(\iota \left(x\right),\ell \left(x\right)\right)}=\sum_{i=1}^{3}{\bar{\theta }}_{v_{i}}\left(x\right)\frac{\iota_{v_{i}}^{*}\left(x\right)\theta_{v_{i}}^{*}\left(x\right)}{\gcd^{*}\left(\iota_{v_{i}}\left(x\right),\ell_{v_{i}}\left(x\right)\right)}v_{i}=\sum_{i=1}^{3}\left(x^{n}-1\right)v_{i}=\left(x^{n}-1\right)\sum_{i=1}^{3}v_{i}=x^{n}-1.$$

Thereby, $\bar{\theta }\left(x\right)=\frac{\left(x^{n}-1\right)\gcd^{*}\left(\iota \left(x\right),\ell \left(x\right)\right)}{\iota^{*}\left(x\right)\theta^{*}\left(x\right)}=\sum_{i=1}^{3}\frac{\left(x^{n}-1\right)\gcd^{*}\left(\iota_{v_{i}}\left(x\right),\ell_{v_{i}}\left(x\right)\right)}{\iota_{v_{i}}^{*}\left(x\right)\theta_{v_{i}}^{*}\left(x\right)}v_{i}$. □

**Remark 5.**  We use the fact that $\deg\left(f^{*}\left(x\right)\right)=\deg\left(f\left(x\right)\right)$ for $f\left(x\right)\in F_{q}\left[x\right]$.

**Proposition 8.**  *Let $C=\langle (\iota (x)|0),(\ell (x)|\theta (x))\rangle =\langle \left(\sum_{i=1}^{3}\iota_{v_{i}}\left(x\right)v_{i}|0\right),\left(\sum_{i=1}^{3}\ell_{v_{i}}\left(x\right)v_{i}|\sum_{i=1}^{3}\theta_{v_{i}}\left(x\right)v_{i}\right)\rangle$ be a double cyclic code of length (m,n) over R, with*

$$C^{⊥}=\langle \left(\bar{\iota }\left(x\right)|0\right),\left(\bar{\ell }\left(x\right)|\bar{\theta }\left(x\right)\right)\rangle =\langle \left(\sum_{i=1}^{3}{\bar{\iota }}_{v_{i}}\left(x\right)v_{i}|0\right),\left(\sum_{i=1}^{3}{\bar{\ell }}_{v_{i}}\left(x\right)v_{i}|\sum_{i=1}^{3}{\bar{\theta }}_{v_{i}}\left(x\right)v_{i}\right)\rangle .$$

Then, $\bar{\ell }\left(x\right)=\rho \left(x\right)\left(\sum_{i=1}^{3}\frac{x^{m}-1}{\iota_{v_{i}}^{*}\left(x\right)}v_{i}\right)$, where

$$\rho \left(x\right)=\left(\sum_{i=1}^{3}-x^{l-\deg\left(\theta_{v_{i}}\left(x\right)\right)+\deg\left(\iota_{v_{i}}\left(x\right)\right)}v_{i}\right){\left(\frac{\iota^{*}\left(x\right)}{\gcd^{*}\left(\iota \left(x\right),\ell \left(x\right)\right)}\right)}^{-1}\mathrm{mod}\frac{\iota^{*}\left(x\right)}{\gcd^{*}\left(\iota \left(x\right),\ell \left(x\right)\right)}.$$

**Proof.**  Since $\left(\bar{\ell }\left(x\right)|\bar{\theta }\left(x\right)\right)\in C^{⊥}$ and (ι(x)|0)∈C, we have

$$\left(\bar{\ell }\left(x\right)|\bar{(}\theta \right)\left(x\right))\circ \left(\iota \left(x\right)|0\right)\equiv 0\;\mathrm{mod}\;\left(x^{l}-1\right)$$

from Lemma 9. Then, we obtain $\bar{\ell }\left(x\right)\iota^{*}\left(x\right)=0\;\mathrm{mod}\;\left(x^{m}-1\right)$ from Lemma 10. Thus, there exists a polynomial ρ(x)∈R[x] such that $\bar{\ell }\left(x\right)=\frac{x^{m}-1}{\iota^{*}\left(x\right)}\rho \left(x\right)=\left(\sum_{i=1}^{3}\frac{x^{m}-1}{\iota_{v_{i}}^{*}\left(x\right)}v_{i}\right)\rho \left(x\right)$. We explain the details of ρ(x) in the following proof.

From Lemma 9, we have $\left(\bar{\ell }\left(x\right)|\bar{\theta }\left(x\right)\right)\circ \left(\ell \left(x\right)|\theta \left(x\right)\right)=0\;\mathrm{mod}\;\left(x^{l}-1\right)$. Writing the concrete expression of $\left(\bar{\ell }\left(x\right)|\bar{\theta }\left(x\right)\right)\circ \left(\ell \left(x\right)|\theta \left(x\right)\right)$, we obtain that

$$\begin{array}{cc} \; & \left(\bar{\ell }\left(x\right)|\bar{\theta }\left(x\right)\right)\circ \left(\ell \left(x\right)|\theta \left(x\right)\right)=\left(\frac{x^{m}-1}{\iota^{*}\left(x\right)}\rho \left(x\right)|\frac{\left(x^{n}-1\right)\gcd^{*}\left(\iota \left(x\right),\ell \left(x\right)\right)}{\iota^{*}\left(x\right)\theta^{*}\left(x\right)}\right)\circ \left(\ell \left(x\right)|\theta \left(x\right)\right)=\sum_{i=1}^{3}\left((\frac{x^{m}-1}{\iota_{v_{i}}^{*}\left(x\right)}\right.\\ & \left.\rho \left(x\right)\omega_{\frac{l}{m}}\left(x^{m}\right)x^{l-1-\deg(\ell_{v_{i}}\left(x\right))}\ell_{v_{i}}^{*}\left(x\right)+\frac{\left(x^{n}-1\right)\gcd_{v_{i}}^{*}\left(\iota \left(x\right),\ell \left(x\right)\right)}{\iota_{v_{i}}^{*}\left(x\right)\theta_{v_{i}}^{*}\left(x\right)}\omega_{\frac{l}{n}}\left(x^{n}\right)x^{l-1-\deg(\theta_{v_{i}}\left(x\right))}\theta_{v_{i}}^{*}\left(x\right)\right)v_{i}. \end{array}$$

And $\left(x^{m}-1\right)\omega_{\frac{l}{m}}\left(x^{m}\right)=x^{l}-1$, $\left(x^{n}-1\right)\omega_{\frac{l}{n}}\left(x^{n}\right)=x^{l}-1$, we have

$$\sum_{i=1}^{3}\frac{\left(x^{l}-1\right)\gcd_{v_{i}}^{*}\left(\iota \left(x\right),\ell \left(x\right)\right)}{\iota_{v_{i}}^{*}\left(x\right)}\left(\rho_{v_{i}}\left(x\right)x^{l-\deg(\ell_{v_{i}}\left(x\right))}\frac{\ell_{v_{i}}^{*}\left(x\right)}{\gcd_{v_{i}}^{*}\left(\iota \left(x\right),\ell \left(x\right)\right)}+x^{l-\deg(\theta_{v_{i}}\left(x\right))-1}\right)v_{i}=0\mathrm{mod}\left(x^{l}-1\right).$$

This reveals that

$$\sum_{i=1}^{3}\frac{x^{l}-1}{\iota_{v_{i}}^{*}\left(x\right)/\gcd_{v}^{*}\left(\iota \left(x\right),\ell \left(x\right)\right)}\left(\rho_{v_{i}}\left(x\right)x^{l-\deg(\ell_{v_{i}}\left(x\right))}\frac{\ell_{v_{i}}^{*}\left(x\right)}{\gcd_{v_{i}}^{*}\left(\iota \left(x\right),\ell \left(x\right)\right)}+x^{l-\deg(\theta_{v_{i}}\left(x\right))-1}\right)v_{i}=0\mathrm{mod}\left(x^{l}-1\right).$$

Set $\tilde{\iota }\left(x\right)=\frac{\iota (x)}{\gcd(\iota (x),\ell (x))},\tilde{\ell }\left(x\right)=\frac{\ell (x)}{\gcd(\iota (x),\ell (x))}$; hence,

$$\sum_{i=1}^{3}\frac{x^{l}-1}{{\tilde{\iota }}_{v_{i}}^{*}\left(x\right)}\left(\rho_{v_{i}}\left(x\right)x^{l-\deg(\ell_{v_{i}}\left(x\right))}{\tilde{\ell }}_{v_{i}}^{*}\left(x\right)+x^{l-\deg(\theta_{v_{i}}\left(x\right))-1}\right)v_{i}=0\;\mathrm{mod}\;\left(x^{l}-1\right).$$

Therefore,

$$\sum_{i=1}^{3}\left(\rho_{v_{i}}\left(x\right)x^{l-\deg(\ell_{v_{i}}\left(x\right))}{\tilde{\ell }}_{v_{i}}^{*}\left(x\right)+x^{l-\deg(\theta_{v_{i}}\left(x\right))-1}\right)v_{i}=0\;\mathrm{mod}\;\left(x^{l}-1\right)$$

or

$$\sum_{i=1}^{3}\left(\rho_{v_{i}}\left(x\right)x^{l-\deg(\ell_{v_{i}}\left(x\right))}{\tilde{\ell }}_{v_{i}}^{*}\left(x\right)+x^{l-\deg(\theta_{v_{i}}\left(x\right))-1}\right)v_{i}=0\;\mathrm{mod}\;\left({\tilde{\iota }}^{*}\left(x\right)\right).$$

Since the former can be deduced the latter by reason of ${\tilde{\iota }}^{*}\left(x\right)|\left(x^{l}-1\right)$, we can assume that

$$\sum_{i=1}^{3}\left(\rho_{v_{i}}\left(x\right)x^{l-\deg(\ell_{v_{i}}\left(x\right))}{\tilde{\ell }}_{v_{i}}^{*}\left(x\right)+x^{l-\deg(\theta_{v_{i}}\left(x\right))-1}\right)v_{i}=0\;\mathrm{mod}\;\left({\tilde{\iota }}^{*}\left(x\right)\right).$$

From the fixing of $\tilde{\iota }\left(x\right)$ and $\tilde{\ell }\left(x\right)$, it is obvious that $\gcd(\tilde{\iota }\left(x\right),\tilde{\ell }\left(x\right))=1$. Furthermore, $x^{l}=1\;\mathrm{mod}\;\iota^{*}\left(x\right)$. Then, ${\tilde{\ell }}^{*}\left(x\right)$ is an invertible element modulo ${\tilde{\iota }}^{*}\left(x\right)$. Consequently, we have

$$\rho \left(x\right)=\left(\sum_{i=1}^{3}-x^{l-\deg\left(\theta_{v_{i}}\left(x\right)\right)+\deg\left(\iota_{v_{i}}\left(x\right)\right)}v_{i}\right){\left(\frac{\ell^{*}\left(x\right)}{\gcd^{*}\left(\iota \left(x\right),\ell \left(x\right)\right)}\right)}^{-1}\mathrm{mod}\frac{\iota^{*}\left(x\right)}{\gcd^{*}\left(\iota \left(x\right),\ell \left(x\right)\right)}.$$

□

Summarizing several propositions and lemmas, we obtain the second primary theorem of this article.

**Theorem 2.**  *Let C be an R-double cyclic code of length (m,n), and set $C,C^{⊥}$ to have the forms of*

$$\left\{\begin{array}{cc} C & =\langle (\iota (x)|0),(\ell (x)|\theta (x))\rangle =\langle \left(\sum_{i=1}^{3}\iota_{v_{i}}\left(x\right)v_{i}|0\right),\left(\sum_{i=1}^{3}\ell_{v_{i}}\left(x\right)v_{i}|\sum_{i=1}^{3}\theta_{v_{i}}\left(x\right)v_{i}\right)\rangle ,\\ C^{⊥} & =\langle \left(\bar{\iota }\left(x\right)|0\right),\left(\bar{\ell }\left(x\right)|\bar{\theta }\left(x\right)\right)\rangle =\langle \left(\sum_{i=1}^{3}{\bar{\iota }}_{v_{i}}\left(x\right)v_{i}|0\right),\left(\sum_{i=1}^{3}{\bar{\ell }}_{v_{i}}\left(x\right)v_{i}|\sum_{i=1}^{3}{\bar{\theta }}_{v_{i}}\left(x\right)v_{i}\right)\rangle . \end{array}\right.$$

*Then,*

(1) $\bar{\iota }\left(x\right)=\sum_{i=1}^{3}\frac{x^{m}-1}{\gcd^{*}\left(\iota_{v_{i}}\left(x\right),\ell_{v_{i}}\left(x\right)\right)}v_{i}$.

(2) $\bar{\theta }\left(x\right)=\sum_{i=1}^{3}\frac{\left(x^{n}-1\right)\gcd^{*}\left(\iota_{v_{i}}\left(x\right),\ell_{v_{i}}\left(x\right)\right)}{\iota_{v_{i}}^{*}\left(x\right)\theta_{v_{i}}^{*}\left(x\right)}v_{i}$.

(3) $\bar{\ell }\left(x\right)=\rho \left(x\right)\sum_{i=1}^{3}\frac{x^{m}-1}{\iota_{v_{i}}^{*}\left(x\right)}v_{i}$, where

$$\left\{\begin{array}{c} \rho (x)=0\;\mathrm{if}\;C\;\mathrm{is}\;\mathrm{separable},\;\mathrm{or}\;\mathrm{otherwise}\\ \rho \left(x\right)=\left(\sum_{i=1}^{3}-x^{l-\deg\left(\theta_{v_{i}}\left(x\right)\right)+\deg\left(\iota_{v_{i}}\left(x\right)\right)}v_{i}\right){\left(\frac{\iota^{*}\left(x\right)}{\gcd^{*}\left(\iota \left(x\right),\ell \left(x\right)\right)}\right)}^{-1}\mathrm{mod}\frac{\iota^{*}\left(x\right)}{\gcd^{*}\left(\iota \left(x\right),\ell \left(x\right)\right)}. \end{array}\right.$$

## 6. Examples of Optimal Codes

In this section, we provide some examples of double cyclic codes over $F_{q}+vF_{q}+v^{2}F_{q}$ with $v^{3}=v$. According to [22], the codes that we present in this section have optimal parameters.

**Example 1.**  *Let $\iota \left(x\right)=x+v^{2}+2v+2$, $\ell \left(x\right)=v^{2}+v+2$ and θ(x)=1, where $F_{q}=F_{3}$, m=n=4.*

*Since $\iota \left(x\right)=x+2v^{2}+v+1=\left(x+1\right)v_{1}+\left(x+1\right)v_{2}+\left(x+2\right)v_{3}$, $\ell \left(x\right)=v^{2}+v+2=2v_{1}+v_{2}+2v_{3}$ and $\theta \left(x\right)=1=v_{1}+v_{2}+v_{3}$. This means that*

$$C_{v_{1}}=\langle (x+2|0),(2|1)\rangle ,C_{v_{2}}=\langle (x+2|0),(1|1)\rangle ,C_{v_{3}}=\langle (x+1|0),(2|1)\rangle .$$

*Therefore, $C_{v_{i}},i=1,2,3$ have the following generator matrices:*

$$G_{1}=\left(\begin{array}{cc} \begin{array}{ccccc} 2 & 1 & 0 & 0 & \\ 0 & 2 & 1 & 0 & \\ 0 & 0 & 2 & 1 & \\ 2 & 0 & 0 & 0 & \\ 0 & 2 & 0 & 0 & \\ 0 & 0 & 2 & 0 & \\ 0 & 0 & 0 & 2 & \end{array}| & \begin{array}{ccccc} 0 & 0 & 0 & 0 & \\ 0 & 0 & 0 & 0 & \\ 0 & 0 & 0 & 0 & \\ 1 & 0 & 0 & 0 & \\ 0 & 1 & 0 & 0 & \\ 0 & 0 & 1 & 0 & \\ 0 & 0 & 0 & 1 & \end{array} \end{array}\right),G_{2}=\left(\begin{array}{cc} \begin{array}{ccccc} 2 & 1 & 0 & 0 & \\ 0 & 2 & 1 & 0 & \\ 0 & 0 & 2 & 1 & \\ 1 & 0 & 0 & 0 & \\ 0 & 1 & 0 & 0 & \\ 0 & 0 & 1 & 0 & \\ 0 & 0 & 0 & 1 & \end{array}| & \begin{array}{ccccc} 0 & 0 & 0 & 0 & \\ 0 & 0 & 0 & 0 & \\ 0 & 0 & 0 & 0 & \\ 1 & 0 & 0 & 0 & \\ 0 & 1 & 0 & 0 & \\ 0 & 0 & 1 & 0 & \\ 0 & 0 & 0 & 1 & \end{array} \end{array}\right),G_{3}=\left(\begin{array}{cc} \begin{array}{ccccc} 1 & 1 & 0 & 0 & \\ 0 & 1 & 1 & 0 & \\ 0 & 0 & 1 & 1 & \\ 2 & 0 & 0 & 0 & \\ 0 & 2 & 0 & 0 & \\ 0 & 0 & 2 & 0 & \\ 0 & 0 & 0 & 2 & \end{array}| & \begin{array}{ccccc} 0 & 0 & 0 & 0 & \\ 0 & 0 & 0 & 0 & \\ 0 & 0 & 0 & 0 & \\ 1 & 0 & 0 & 0 & \\ 0 & 1 & 0 & 0 & \\ 0 & 0 & 1 & 0 & \\ 0 & 0 & 0 & 1 & \end{array} \end{array}\right).$$

It is easy to see that all three codes $C_{v_{i}},i=1,2,3$ are MDS codes with parameter [8,7,2] over $F_{3}$. Therefore, $C=C_{v_{1}}v_{1}⊕C_{v_{2}}v_{2}⊕C_{v_{3}}v_{3}$ is a ${\left[24,21,2\right]}_{3}$ code. From [22], we know that this is an optimal code over $F_{3}$.

**Example 2.**  *Let $\iota \left(x\right)=x+2v^{2}+v+1$, $\ell \left(x\right)=2v^{2}+2$ and $\theta \left(x\right)=2v^{2}+v+1$, where $F_{q}=F_{3}$, m=n=6.*

*Since $\iota \left(x\right)=x+2v^{2}+v+1=\left(x+1\right)v_{1}+\left(x+1\right)v_{2}+\left(x+2\right)v_{3}$, $\ell \left(x\right)=v^{2}+v+2=2v_{1}+v_{2}+v_{3}$ and $\theta \left(x\right)=2v^{2}+v+1=v_{1}+v_{2}+2v_{3}$, this means that*

$$C_{v_{1}}=\langle (x+1|0),(2|1)\rangle ,C_{v_{2}}=\langle (x+1|0),(1|1)\rangle ,C_{v_{3}}=\langle (x+2|0),(1|2)\rangle .$$

*Therefore, $C_{v_{i}},i=1,2,3$ have the following generator matrices:*

$$G_{1}=\left(\begin{array}{cc} \begin{array}{ccccccc} 1 & 1 & 0 & 0 & 0 & 0 & \\ 0 & 1 & 1 & 0 & 0 & 0 & \\ 0 & 0 & 1 & 1 & 0 & 0 & \\ 0 & 0 & 0 & 1 & 1 & 0 & \\ 0 & 0 & 0 & 0 & 1 & 1 & \\ 2 & 0 & 0 & 0 & 0 & 0 & \\ 0 & 2 & 0 & 0 & 0 & 0 & \\ 0 & 0 & 2 & 0 & 0 & 0 & \\ 0 & 0 & 0 & 2 & 0 & 0 & \\ 0 & 0 & 0 & 0 & 2 & 0 & \\ 0 & 0 & 0 & 0 & 0 & 2 & \end{array}| & \begin{array}{ccccccc} 0 & 0 & 0 & 0 & 0 & 0 & \\ 0 & 0 & 0 & 0 & 0 & 0 & \\ 0 & 0 & 0 & 0 & 0 & 0 & \\ 0 & 0 & 0 & 0 & 0 & 0 & \\ 0 & 0 & 0 & 0 & 0 & 0 & \\ 1 & 0 & 0 & 0 & 0 & 0 & \\ 0 & 1 & 0 & 0 & 0 & 0 & \\ 0 & 0 & 1 & 0 & 0 & 0 & \\ 0 & 0 & 0 & 1 & 0 & 0 & \\ 0 & 0 & 0 & 0 & 1 & 0 & \\ 0 & 0 & 0 & 0 & 0 & 1 & \end{array} \end{array}\right),$$

$$G_{2}=\left(\begin{array}{cc} \begin{array}{ccccccc} 1 & 1 & 0 & 0 & 0 & 0 & \\ 0 & 1 & 1 & 0 & 0 & 0 & \\ 0 & 0 & 1 & 1 & 0 & 0 & \\ 0 & 0 & 0 & 1 & 1 & 0 & \\ 0 & 0 & 0 & 0 & 1 & 1 & \\ 1 & 0 & 0 & 0 & 0 & 0 & \\ 0 & 1 & 0 & 0 & 0 & 0 & \\ 0 & 0 & 1 & 0 & 0 & 0 & \\ 0 & 0 & 0 & 1 & 0 & 0 & \\ 0 & 0 & 0 & 0 & 1 & 0 & \\ 0 & 0 & 0 & 0 & 0 & 1 & \end{array}| & \begin{array}{ccccccc} 0 & 0 & 0 & 0 & 0 & 0 & \\ 0 & 0 & 0 & 0 & 0 & 0 & \\ 0 & 0 & 0 & 0 & 0 & 0 & \\ 0 & 0 & 0 & 0 & 0 & 0 & \\ 0 & 0 & 0 & 0 & 0 & 0 & \\ 1 & 0 & 0 & 0 & 0 & 0 & \\ 0 & 1 & 0 & 0 & 0 & 0 & \\ 0 & 0 & 1 & 0 & 0 & 0 & \\ 0 & 0 & 0 & 1 & 0 & 0 & \\ 0 & 0 & 0 & 0 & 1 & 0 & \\ 0 & 0 & 0 & 0 & 0 & 1 & \end{array} \end{array}\right)$$

*and*

$$G_{3}=\left(\begin{array}{cc} \begin{array}{ccccccc} 2 & 1 & 0 & 0 & 0 & 0 & \\ 0 & 2 & 1 & 0 & 0 & 0 & \\ 0 & 0 & 2 & 1 & 0 & 0 & \\ 0 & 0 & 0 & 2 & 1 & 0 & \\ 0 & 0 & 0 & 0 & 2 & 1 & \\ 1 & 0 & 0 & 0 & 0 & 0 & \\ 0 & 1 & 0 & 0 & 0 & 0 & \\ 0 & 0 & 1 & 0 & 0 & 0 & \\ 0 & 0 & 0 & 1 & 0 & 0 & \\ 0 & 0 & 0 & 0 & 1 & 0 & \\ 0 & 0 & 0 & 0 & 0 & 1 & \end{array}| & \begin{array}{ccccccc} 0 & 0 & 0 & 0 & 0 & 0 & \\ 0 & 0 & 0 & 0 & 0 & 0 & \\ 0 & 0 & 0 & 0 & 0 & 0 & \\ 0 & 0 & 0 & 0 & 0 & 0 & \\ 0 & 0 & 0 & 0 & 0 & 0 & \\ 2 & 0 & 0 & 0 & 0 & 0 & \\ 0 & 2 & 0 & 0 & 0 & 0 & \\ 0 & 0 & 2 & 0 & 0 & 0 & \\ 0 & 0 & 0 & 2 & 0 & 0 & \\ 0 & 0 & 0 & 0 & 2 & 0 & \\ 0 & 0 & 0 & 0 & 0 & 2 & \end{array} \end{array}\right).$$

From Magma, we know that all three codes $C_{v_{i}},i=1,2,3$ are MDS codes with parameter [12,11,2] over $F_{3}$. Consequently, $C=C_{v_{1}}v_{1}⊕C_{v_{2}}v_{2}⊕C_{v_{3}}v_{3}$ is a ${\left[36,33,2\right]}_{3}$ code. According to [22], this is also an optimal code over $F_{3}$.

**Example 3.**  *Let $F_{q}=F_{3}$, m=n=7. At the same time, let ι(x)=x+2, $\ell \left(x\right)=v^{2}+v+2$ and $\theta \left(x\right)=\left(2v^{2}+v\right)x+2v^{2}+v+1$.*

*Since $\iota \left(x\right)=x+2=\left(x+2\right)v_{1}+\left(x+2\right)v_{2}+\left(x+2\right)v_{3}$, $\ell \left(x\right)=v^{2}+v+2=2v_{1}+v_{2}+v_{3}$ and $\theta \left(x\right)=\left(2v^{2}+v\right)x+2v^{2}+v+1=v_{1}+v_{2}+\left(x+2\right)v_{3}$. This means that*

$$C_{v_{1}}=\langle (x+2|0),(2|1)\rangle ,C_{v_{2}}=\langle (x+2|0),(1|1)\rangle ,C_{v_{3}}=\langle (x+2|0),(1|x+2)\rangle .$$

*Therefore, $C_{v_{i}},i=1,2,3$ have the following generator matrices:*

$$G_{1}=\left(\begin{array}{cc} \begin{array}{cccccccc} 2 & 1 & 0 & 0 & 0 & 0 & 0 & \\ 0 & 2 & 1 & 0 & 0 & 0 & 0 & \\ 0 & 0 & 2 & 1 & 0 & 0 & 0 & \\ 0 & 0 & 0 & 2 & 1 & 0 & 0 & \\ 0 & 0 & 0 & 0 & 2 & 1 & 0 & \\ 0 & 0 & 0 & 0 & 0 & 2 & 1 & \\ 2 & 0 & 0 & 0 & 0 & 0 & 0 & \\ 0 & 2 & 0 & 0 & 0 & 0 & 0 & \\ 0 & 0 & 2 & 0 & 0 & 0 & 0 & \\ 0 & 0 & 0 & 2 & 0 & 0 & 0 & \\ 0 & 0 & 0 & 0 & 2 & 0 & 0 & \\ 0 & 0 & 0 & 0 & 0 & 2 & 0 & \\ 0 & 0 & 0 & 0 & 0 & 0 & 2 & \end{array}| & \begin{array}{cccccccc} 0 & 0 & 0 & 0 & 0 & 0 & 0 & \\ 0 & 0 & 0 & 0 & 0 & 0 & 0 & \\ 0 & 0 & 0 & 0 & 0 & 0 & 0 & \\ 0 & 0 & 0 & 0 & 0 & 0 & 0 & \\ 0 & 0 & 0 & 0 & 0 & 0 & 0 & \\ 0 & 0 & 0 & 0 & 0 & 0 & 0 & \\ 1 & 0 & 0 & 0 & 0 & 0 & 0 & \\ 0 & 1 & 0 & 0 & 0 & 0 & 0 & \\ 0 & 0 & 1 & 0 & 0 & 0 & 0 & \\ 0 & 0 & 0 & 1 & 0 & 0 & 0 & \\ 0 & 0 & 0 & 0 & 1 & 0 & 0 & \\ 0 & 0 & 0 & 0 & 0 & 1 & 0 & \\ 0 & 0 & 0 & 0 & 0 & 0 & 1 & \end{array} \end{array}\right),$$

$$G_{2}=\left(\begin{array}{cc} \begin{array}{cccccccc} 2 & 1 & 0 & 0 & 0 & 0 & 0 & \\ 0 & 2 & 1 & 0 & 0 & 0 & 0 & \\ 0 & 0 & 2 & 1 & 0 & 0 & 0 & \\ 0 & 0 & 0 & 2 & 1 & 0 & 0 & \\ 0 & 0 & 0 & 0 & 2 & 1 & 0 & \\ 0 & 0 & 0 & 0 & 0 & 2 & 1 & \\ 1 & 0 & 0 & 0 & 0 & 0 & 0 & \\ 0 & 1 & 0 & 0 & 0 & 0 & 0 & \\ 0 & 0 & 1 & 0 & 0 & 0 & 0 & \\ 0 & 0 & 0 & 1 & 0 & 0 & 0 & \\ 0 & 0 & 0 & 0 & 1 & 0 & 0 & \\ 0 & 0 & 0 & 0 & 0 & 1 & 0 & \\ 0 & 0 & 0 & 0 & 0 & 0 & 1 & \end{array}| & \begin{array}{cccccccc} 0 & 0 & 0 & 0 & 0 & 0 & 0 & \\ 0 & 0 & 0 & 0 & 0 & 0 & 0 & \\ 0 & 0 & 0 & 0 & 0 & 0 & 0 & \\ 0 & 0 & 0 & 0 & 0 & 0 & 0 & \\ 0 & 0 & 0 & 0 & 0 & 0 & 0 & \\ 0 & 0 & 0 & 0 & 0 & 0 & 0 & \\ 1 & 0 & 0 & 0 & 0 & 0 & 0 & \\ 0 & 1 & 0 & 0 & 0 & 0 & 0 & \\ 0 & 0 & 1 & 0 & 0 & 0 & 0 & \\ 0 & 0 & 0 & 1 & 0 & 0 & 0 & \\ 0 & 0 & 0 & 0 & 1 & 0 & 0 & \\ 0 & 0 & 0 & 0 & 0 & 1 & 0 & \\ 0 & 0 & 0 & 0 & 0 & 0 & 1 & \end{array} \end{array}\right)$$

*and*

$$G_{3}=\left(\begin{array}{cc} \begin{array}{cccccccc} 2 & 1 & 0 & 0 & 0 & 0 & 0 & \\ 0 & 2 & 1 & 0 & 0 & 0 & 0 & \\ 0 & 0 & 2 & 1 & 0 & 0 & 0 & \\ 0 & 0 & 0 & 2 & 1 & 0 & 0 & \\ 0 & 0 & 0 & 0 & 2 & 1 & 0 & \\ 0 & 0 & 0 & 0 & 0 & 2 & 1 & \\ 1 & 0 & 0 & 0 & 0 & 0 & 0 & \\ 0 & 1 & 0 & 0 & 0 & 0 & 0 & \\ 0 & 0 & 1 & 0 & 0 & 0 & 0 & \\ 0 & 0 & 0 & 1 & 0 & 0 & 0 & \\ 0 & 0 & 0 & 0 & 1 & 0 & 0 & \\ 0 & 0 & 0 & 0 & 0 & 1 & 0 & \end{array}| & \begin{array}{cccccccc} 0 & 0 & 0 & 0 & 0 & 0 & 0 & \\ 0 & 0 & 0 & 0 & 0 & 0 & 0 & \\ 0 & 0 & 0 & 0 & 0 & 0 & 0 & \\ 0 & 0 & 0 & 0 & 0 & 0 & 0 & \\ 0 & 0 & 0 & 0 & 0 & 0 & 0 & \\ 0 & 0 & 0 & 0 & 0 & 0 & 0 & \\ 2 & 1 & 0 & 0 & 0 & 0 & 0 & \\ 0 & 2 & 1 & 0 & 0 & 0 & 0 & \\ 0 & 0 & 2 & 1 & 0 & 0 & 0 & \\ 0 & 0 & 0 & 2 & 1 & 0 & 0 & \\ 0 & 0 & 0 & 0 & 2 & 1 & 0 & \\ 0 & 0 & 0 & 0 & 0 & 2 & 1 & \end{array} \end{array}\right).$$

According to Magma, we know that $C_{v_{1}}$ and $C_{v_{2}}$ are MDS codes with parameters [14,13,2] over $F_{3}$, while $C_{v_{3}}$ has the parameter [14,12,2] over $F_{3}$. Then, $C=C_{v_{1}}v_{1}⊕C_{v_{2}}v_{2}⊕C_{v_{3}}v_{3}$ is a ${\left[42,38,2\right]}_{3}$ code in the same way. According to [22], this is also an optimal code over $F_{3}$.

These examples show that we can use some MDS codes with small lengths to construct an optimal code with a considerable length.

## 7. Conclusions

In this paper, we analyzed the algebraic structure of double cyclic codes over $F_{q}+vF_{q}+v^{2}F_{q}$ with $v^{3}=v$. We provided the generator polynomials of these codes, and give their generating matrix forms. Furthermore, we discussed the quantitative relationship between the double cyclic codes and their duals. Finally, we determined the relationship between the generators of double cyclic codes and their duals. As an application, we obtained some optimal codes with longer length by using MDS codes with shorter lengths. Last year, Biswas and Maheshanand Bhaintwal considered a new construction of quantum codes from quasi-cyclic codes over finite fields in [23]. Meanwhile, Mohammad Ashraf et al. considered quantum codes from cyclic codes over a mixed alphabet structure in [24]. Therefore, as a special case of generalized quasi-cyclic codes, we can also considered the construction of quantum codes in the future. 

## Data Availability

Data available on request and not publicly accessible due to ownership restrictions.
